# Fibronectin-guided immune–stromal cell crosstalk promotes angiogenesis in pulp–dentin complex regeneration

**DOI:** 10.1038/s12276-026-01863-4

**Published:** 2026-10-06

**Authors:** Sol Jeong, So Young Park, Hyemin Ku, Byeong Soo Eom, Dohyun Kim, Jin Man Kim

**Affiliations:** 1 Department of Oral Microbiology and Immunology, School of Dentistry and Dental Research Institute, Seoul National University, Seoul, 08826 Republic of Korea; 2 Department of Conservative Dentistry and Oral Science Research Center, Yonsei University College of Dentistry, 50-1 Yonsei-ro, Seodaemun-gu, Seoul, 03722 Republic of Korea; 3 Dental Multiomics Center, School of Dentistry and Dental Research Institute, Seoul National University, Gwanak-ro 1, Gwanak-gu, Seoul, 08826 Republic of Korea; 4 Innovative Pharmaceutical Sciences Program, College of Transdisciplinary Innovations, Seoul National University, Gwanak-ro 1, Gwanak-gu, Seoul, 08826 Republic of Korea

**Keywords:** Extracellular signalling molecules, Stem-cell niche

## Abstract

Macrophages are multifunctional immune cells that play crucial roles in immune regulation and tissue regeneration by interacting with various cell types. Although macrophage–stromal cell interactions are widely recognized as essential for tissue regeneration and homeostasis, their precise mechanisms in the pulp–dentin complex, a key functional unit for tooth maintenance, remain incompletely understood. In this study, we investigated the interactions between macrophages and dental pulp stromal cells (DPSCs) within a dental pulp microenvironment in response to dental caries infection. As caries progressed, macrophage–DPSC interactions became increasingly prominent. Whole-transcriptome analysis revealed that these interactions are mediated by fibronectin–integrin engagement, which induces a phenotypic shift in macrophages from pro-inflammatory to pro-angiogenic, mediated by the upregulation of angiogenic chemokines. Integrated analysis of a single-cell RNA sequencing dataset from human pulp tissue further confirmed that caries progression enhanced macrophage-driven angiogenic signalling in the endothelial cell population. Moreover, the altered secretome resulting from the macrophage–DPSC co-culture significantly enhanced in vitro endothelial tube formation and survival, indicating a strong angiogenic potential conducive to tissue regeneration. In a preclinical pulp revascularization model, the application of fibronectin during revascularization enhanced neovascularization, root dentin formation, apical maturation and organized pulp–dentin-like tissue formation. Collectively, these findings indicate that fibronectin is a key regulator that reprogrammes the damaged pulp environment into a pro-angiogenic and regenerative niche through coordinated immune–stromal interactions. This study provides a promising direction for the development of extracellular matrix-based therapeutic strategies in regenerative dentistry.

## Fibronectin Reprogrammes Macrophages for Tooth Regeneration

Dental caries often leads to severe conditions such as pulpitis and apical periodontitis if untreated. Current treatments, although effective in repairing structural damage, fail to regenerate natural tooth structures. This study explores macrophage interactions within the dental pulp microenvironment, focusing on their role in tissue regeneration. Researchers used a combination of spatial tissue analysis and co-culture systems to investigate macrophage–dental pulp stromal cell interactions, revealing that fibronectin–integrin engagement activates NFκB signalling, promoting angiogenesis. The study found that fibronectin treatment in a canine model enhanced vascularization, root dentin formation and organized pulp–dentin-like tissue formation. These findings suggest the potential of fibronectin in regenerative dentistry, offering a pathway for developing therapies that promote natural tooth regeneration and improve treatment outcomes. Future research could explore the synergistic effects of fibronectin with other bioactive molecules to enhance regenerative therapies further.

This summary was initially drafted using artificial intelligence, then revised and fact-checked by the author.

## Introduction

Dental caries is the most prevalent oral disease, with over 2.5 billion people affected by untreated lesions in their permanent teeth^1^. If left untreated, these hard-tissue lesions can progress to involve the dental pulp and periapical tissues, leading to pulpitis and apical periodontitis. A recent meta-analysis found apical periodontitis in approximately 52% of individuals worldwide, underscoring the widespread impact of these conditions on public health^2^. The primary goal of dental treatment is to restore function and aesthetics while preserving natural teeth. Current management of dental caries relies on the replacement of hard-tissue defects with artificial materials such as composites or ceramics. Although these restorative treatments can successfully repair structural damage, they do not reproduce the natural tooth structure and are prone to failure over time owing to degradation and microleakage^3^. For pulpal and periapical diseases, root canal treatment is a standard procedure that mechanochemically removes infected pulpal tissues from the root canals. However, it devitalizes the tooth, compromising root strength and lacking the capacity for further dentin formation^4^. Consequently, modern dentistry is increasingly pursuing tissue regeneration-based strategies for the physiological recovery of damaged tissues.

Various methodologies have been introduced for dental hard-tissue regeneration, including the use of dental pulp stromal cells (DPSCs), scaffold materials and bioactive factors^5^. However, achieving complete functional regeneration remains challenging. The outcomes of regenerative procedures remain inconsistent and unpredictable, with the true regeneration of a functional pulp–dentin complex rarely observed^6^. Newly formed tissue often consists of fibrous connective tissue intermixed with calcified deposits rather than organized pulp tissue with properly differentiated odontoblasts capable of forming tubular dentin^7,8^. These limitations highlight the critical need for a deeper understanding of the biological mechanisms underlying pulp–dentin complex regeneration to improve therapeutic predictability and efficacy.

Macrophages are the central effectors of the innate immune system and function as phagocytes, playing a pivotal role in the detection, elimination and resolution of pathogenic threats. As frontline immune sentinels, they recognize pathogen-associated molecular patterns (PAMPs) via pattern recognition receptors (PRRs), including Toll-like receptors (TLRs), and initiate robust inflammatory responses^9^. Macrophages are indispensable mediators of tissue remodelling and regeneration. Following injury, cells undergo phenotypic transitions that support the sequential phases of wound healing. Macrophages secrete various trophic factors such as transforming growth factor-beta (TGFβ), vascular endothelial growth factor (VEGF) and insulin-like growth factor-1 (IGF-1), which regulate the proliferation, migration and differentiation of resident stromal and progenitor cells. Additionally, their ability to promote angiogenesis and modulate extracellular matrix (ECM) remodelling contributes to the establishment of a regenerative microenvironment conducive to tissue repair^10–12^.

Based on the concept of cell-to-cell communication, macrophages are also recognized as key regulators of tissue regeneration through dynamic interactions with various cell types. Macrophages play a direct regenerative role by forming transient niches at injury sites, such as in the skeletal muscle, where they promote muscle stem cell proliferation^13^. Under homeostatic conditions, macrophages and stromal cells maintain tissue integrity via stable reciprocal signalling that supports survival and function^14^. During osteogenesis, macrophage–stromal cell crosstalk modulates immune responses and enhances stromal cell proliferation and osteogenic differentiation, positioning macrophages as key coordinators of tissue repair and remodelling^15,16^. Therefore, understanding the interactions of macrophages with various cell types is critical across diverse fields of regenerative research, including hard-tissue regeneration.

The pulp–dentin complex functions as an integrated unit to maintain the structural integrity of the teeth. This integrated system depends on the reciprocal interactions between various cellular components that maintain dental pulp homeostasis. Following an infectious insult from dental caries or trauma, the lesion progressively advances through the enamel and dentin, eventually involving the pulpal tissue, in the absence of appropriate intervention. Structural or pathological changes at the pulp–dentin interface generate a dynamic network of interdependent relationships among odontoblasts, stromal cells and immune cells^17^. Odontoblasts, located along the periphery of the pulp space, with their processes extending into the dentinal tubules, serve as essential sentinels in the defence mechanisms of the pulp–dentin complex. This unique cell type detects and responds to external stimuli by producing tertiary dentin as a defence barrier, thereby contributing to the preservation of pulp vitality and tooth integrity^18^. Fibroblasts, the most abundant stromal cell type within the pulp, are responsible for the production and maintenance of the ECM^19^. As a unique stromal cell type, DPSCs are particularly important because of their regenerative and immunomodulatory properties^20^. DPSCs possess the ability to differentiate into odontoblast-like cells and contribute to reparative dentin formation after injury^8^. Because of their multipotency, DPSCs are attractive targets for the development of therapeutic strategies for regenerative endodontic treatments.

Immune cells are also essential contributors to the defence and regeneration of the pulp–dentin complex. Macrophages are the critical regulators of inflammation and pulp tissue repair. In bacteria-induced pulpitis, resident macrophages, along with recruited neutrophils and monocytes, comprise the predominant immune-cell population within the inflamed pulp tissue^21^. As caries progresses, macrophages accumulate near the odontoblast layer^22^, where they respond to infection-associated signals and undergo phenotypic polarization^23^. This dynamic shift in functional state modulates the local immune environment and profoundly influences the course of tissue regeneration^24^. Macrophages contribute to early wound healing in reversible pulpitis and act as key mediators of reparative dentin formation by activating DPSC and facilitating anti-inflammatory responses during dental repair^25,26^. These responses underscore the importance of macrophages in orchestrating innate immune regulation and maintaining pulpal homeostasis. Despite their diverse roles in pulpal immunity and regeneration, a comprehensive understanding of macrophage interactions in dental pulp, particularly their modes of communication and interacting partners, has not yet been fully elucidated.

In this study, we systematically investigated macrophage interactions within the dental pulp microenvironment and identified the key mediators that modulate intercellular communication. Our findings elucidate the macrophage-mediated signalling networks and provide a foundation for the development of novel therapeutic strategies for regenerative dentistry.

## Materials and methods

### Tube formation assay

Matrigel (Corning, 356231) was aliquoted into 96-well plates (Ibidi, 89646) and incubated at 37 °C for 60 min. Subsequently, cells were counted, resuspended in conditioned medium (CM) and seeded in gel-coated wells. After 16 h of incubation, the cells were examined under a microscope. To quantify angiogenic activity, tube formation was analysed using the Angiogenesis Analyzer plugin in Fiji (ImageJ)^27^.

### Image analysis

Image analysis was performed using Fiji (ImageJ)^28,29^. For quantification of CD68^+^ macrophage distribution, dental pulp regions were segmented into the odontoblast layer, sub-odontoblast layer and central dental pulp based on DAPI staining and morphological landmarks. Regions of interest (ROIs) corresponding to these areas were manually delineated, and the number of CD68^+^ cells per region was quantified using the Analyze Particles function after thresholding. Macrophage density was defined as the number of CD68^+^ cells normalized to the ROI. The proportion of directly contacting cells was defined as the ratio of overlapping area to the total CD68^+^ macrophage area per image. Quantification was performed across multiple fields per sample and averaged for each condition (healthy, moderate and severe caries). For colocalization analysis between integrin α5 clusters and fibronectin, the correlation coefficient was calculated using the Colocalization Threshold plugin in ImageJ. ITGA5 cluster size and distance were determined using the Analyze Particles function, following selection of a ROI at the cell periphery based on fibronectin staining.

### Canine immature tooth pulp revascularization model

All experiments were performed in compliance with the ARRIVE guidelines and other relevant guidelines and regulations. The initial mongrel dog experiment was approved by the Yonsei University Health System Institutional Animal Care and Use Committee (IACUC), Seoul, Republic of Korea (Approval No. P-2021-0130). Additional beagle experiments were performed at Cronex, Suwon, Republic of Korea, and were approved by the Cronex IACUC (Approval No. 202603004). Three independent canine animals were included in this in vivo study: one mongrel dog from the initial experiment and two additional beagles. Immature premolar teeth were assigned to control or fibronectin-treated groups using a split-mouth design. The root-level analysis included 22 roots per group.

The regenerative endodontic procedures were performed in accordance with the American Association of Endodontists (AAE) guidelines^30^. For the initial treatment, pulp extirpation was performed aseptically on the immature premolars under general anaesthesia. Access cavities were prepared using a diamond bur, and complete pulp extirpation was performed to remove all the pulp tissue from the root canals. The canals were irrigated with 5.25% sodium hypochlorite (NaOCl) solution (20 ml/canal, 5 min), and calcium hydroxide paste was used as an intracanal medicament. The access cavities were sealed with temporary restorative material (IRM; Dentsply Sirona, Milford), and periapical radiographs were obtained to confirm the procedures. One week after the initial treatment, revascularization procedures were performed under general anaesthesia. The temporary restoration was removed, and the canals were thoroughly irrigated to remove residual calcium hydroxide. The canals were sequentially irrigated with 5.25% NaOCl (20 ml/canal for 5 min) and 17% ethylenediaminetetraacetic acid (EDTA) solution (20 ml/canal). In the fibronectin-treated group, the canals were soaked in the fibronectin irrigation solution for 10 min as the final irrigation step. The fibronectin irrigation solution was prepared by diluting 1 mg/ml fibronectin solution (Sigma-Aldrich, St. Louis, MO, USA) 1:10 in PBS to achieve a final concentration of 100 μg/ml. After canal drying, apical bleeding was induced by inserting a sterile endodontic file 2 mm beyond the apex to create a blood clot in the canal space. In the control group, a collagen sponge (CollaCote; Zimmer Dental) sliced to approximately 3 mm × 3 mm × 3 mm and soaked in PBS was placed in the coronal portion of the canal, making direct contact with the blood clot. In the fibronectin-treated group, a fibronectin-containing collagen sponge of the same size was prepared by adsorbing the fibronectin solution onto the collagen sponge and placing it in the same position. Both groups received the same collagen sponge scaffold, differing only in the presence or absence of fibronectin. The coronal portion of the canal was sealed with a mineral trioxide aggregate (RetroMTA; BioMTA), and the access cavity was restored using a dental composite (Tetric N Ceram; Ivoclar Vivadent). Periapical radiographs were obtained immediately after the procedure to confirm proper treatment. Eight weeks after the regenerative procedures, the animals were sacrificed under general anaesthesia following standard veterinary protocols. After obtaining periapical radiographs, all treated teeth were carefully extracted and immediately fixed in 10% buffered paraformaldehyde solution. Double-rooted teeth were hemisected into two single roots using a diamond bur for subsequent micro-computed tomography (μCT) and histological analyses. Each root was analysed as an anatomical unit for μCT and histological assessments, and the number of independent animals was reported separately.

For μCT analysis, the mean cross-sectional root dentin area was measured in each root, and apical closure was assessed at the root apex. Roots were classified as showing open or closed apices based on the presence of apical hard-tissue closure on μCT images. Histological evaluation was performed using haematoxylin and eosin (H&E) staining to assess pulp-like tissue organization, odontoblast-like cells, dentinal tubule-like structures and apical hard-tissue closure. Immunohistochemical staining for dentin sialoprotein (DSP) was performed to assess odontoblastic differentiation in newly formed tissues.

### Statistical analysis

Statistical analyses were performed using GraphPad Prism version 10.1.2 and R version 4.3.2. Descriptive statistics are presented as mean ± standard error of the mean (SEM) or mean ± standard deviation (SD) for continuous variables. Group comparisons were performed using one-way analysis of variance or Student’s *t*test, followed by Bonferroni post hoc multiple comparison tests when appropriate. For the in vivo experiment, roots were analysed as anatomical units, and the paired *t*test and Fisher’s exact test were used. A *P* value < 0.05 was considered statistically significant.

## Results

### Identification of macrophage–dental pulp stromal cell interaction response to dental pulp injury

**Fig. 1:**
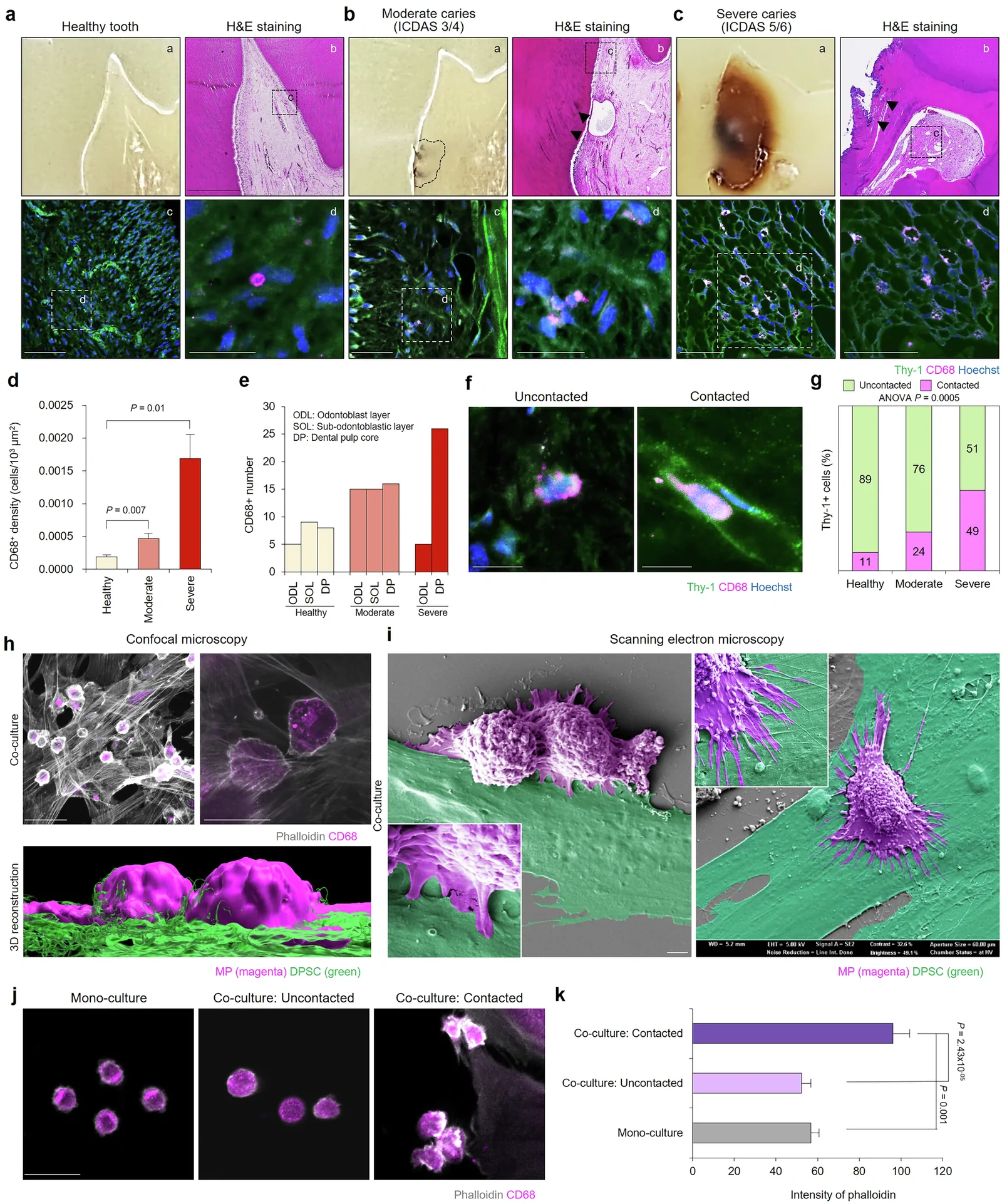
Identification of macrophage and dental pulp stromal cell interactions within pulp tissue. **a–c** Histological and immunofluorescence images of dental pulp tissues from healthy, moderate (dashed outline) and severe caries. **i**, **ii** Light field and haematoxylin and eosin (H&E) staining images; black arrowheads mark infected dentin, rectangles indicate regions enlarged in **iii**. Scale bars: 1000 μm. **iii**, **iv** Immunofluorescence showing Thy-1, CD68 and Hoechst; white rectangles show areas magnified in **iv**. Scale bar: 50 μm. ICDAS, International Caries Detection and Assessment System. **d** Quantification of CD68^+^ macrophages (MPs) in control, moderate and severe caries groups. (*n* = 5; mean ± standard error of the mean (SEM)). **e** Distribution of macrophages across the odontoblast layer (ODL), sub-odontoblastic layer (SOL) and dental pulp core (DP). **f** Representative images showing uncontacted or contacted macrophages with Thy-1^+^ cells. Scale bar: 100 μm. **g** Proportion of uncontacted or contacted macrophages on Thy-1^+^ cells. ANOVA, analysis of variance. **h** (*Upper*) Immunofluorescence images of phalloidin and CD68 under co-culture. (*Lower*) 3D images depicting MPs and dental pulp stromal cells (DPSCs). **i** Electron microscopy images of MPs and DPSCs in co-culture, showing lamellipodia (left) and filopodia-like structures (right). Scale bar: 2 μm. **j** Immunofluorescence images of phalloidin and CD68 in mono-culture, co-culture uncontacted and contacted MPs. **k** Quantification of phalloidin intensity from **j** (*n* = 15; mean ± SEM).

Among the immune cells in the dental pulp, macrophages are particularly significant because of their marked plasticity and essential functions, including modulation of inflammation, promotion of odontoblast differentiation and support of tertiary dentin formation^25^. To elucidate the potential role of macrophages in dental pulp responses to external stimuli, we investigated their spatial distribution within human dental pulp tissues from healthy teeth and teeth with moderate or severe caries (Fig. 1a–c). Quantitative analysis of the immunofluorescence images revealed the characteristic spatial distribution of CD68^+^ macrophages across different stages of caries. Following caries progression, a marked increase in the number of CD68^+^ macrophages was observed (Fig. 1d). In healthy teeth, CD68^+^ macrophages were distributed throughout the entire region of the dental pulp at a low density, including the odontoblast layer, sub-odontoblastic layer and pulp core (Fig. 1a, e). As the caries progressed to a moderate stage, CD68^+^ macrophages showed an increased presence near the odontoblast layer adjacent to the infected dentin and exhibited a more frequent distribution throughout the pulp space (Fig. 1b, e). Severe caries exhibited a markedly increased density of CD68^+^ macrophages, particularly beneath regions of advanced decay, accompanied by widespread infiltration of the dental pulp (Fig. 1c, e). Notably, a considerable portion of CD68^+^ macrophages established intimate contact with Thy-1^+^ human DPSCs (Fig. 1f). Moreover, the proportion of contact between macrophages and DPSCs increased as caries progressed from 11.2% in healthy teeth to 23.7% in moderate and 48.7% in severe caries, suggesting a possible role for immune–stromal interactions in the defensive response of infected pulp tissues (Fig. 1g).

To characterize the interactions between macrophages and DPSCs, we established an in vitro co-culture system using differentiated U937 cells and primary human DPSCs. Under two-dimensional co-culture conditions, the two cell types exhibited extensive contact with each other (Fig. 1h). Reconstructed three-dimensional images confirmed the direct contact between macrophages and DPSCs, providing structural evidence for their functional interactions (Fig. 1h). Scanning electron microscopy images revealed the morphological variability of the macrophages in contact with DPSCs, highlighting the presence of protrusions resembling lamellipodia or filopodia, which facilitate biophysical interactions with DPSCs (Fig. 1i). We compared the filamentous actin (F-actin) patterns of macrophages based on whether they were in contact with DPSCs (Fig. 1j). Macrophages in direct contact with DPSCs exhibited markedly increased F-actin intensity compared with uncontacted macrophages under both mono-culture and co-culture conditions (Fig. 1k). This enhanced actin polymerization was particularly evident at the interface between the macrophages and DPSCs, indicating the involvement of an intercellular adhesion mechanism^31^.

### Fibronectin is a key mediator of macrophage–dental pulp stromal cell interaction

**Fig. 2:**
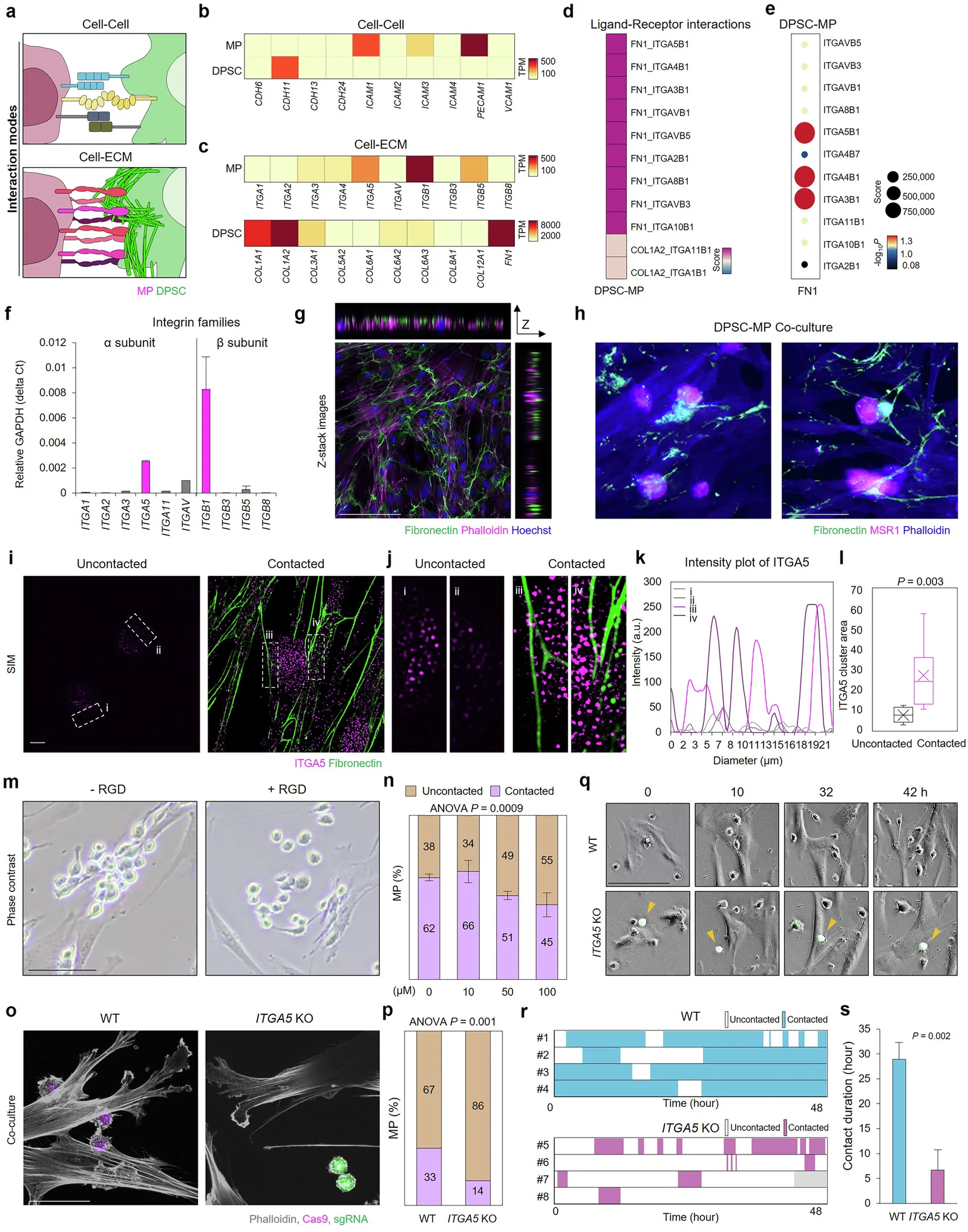
Discovery of fibronectin-mediated intercellular interactions. **a** Scheme of intercellular interactions. **b**, **c** Heat maps of genes related to the intercellular interactions between macrophages (MPs) and dental pulp stromal cells (DPSCs). **d**, **e** Heatmaps and dot plots of integrin–ECM interactions between MPs and DPSCs (CellPhoneDB analysis). **f** mRNA expression of the integrin subfamily in MPs (*n* = 3; mean ± standard error of the mean (SEM)). **g** Z-stack immunofluorescence images of DPSCs showing fibronectin and phalloidin. Scale bar: 100 μm. **h** Immunofluorescence of co-cultures showing fibronectin, phalloidin and MSR1. Scale bar: 100 μm. **i** Structured illumination microscopy (SIM) images showing integrin α5 and fibronectin in macrophages. Scale bar: 5 µm. **j** Magnified view of the white dashed regions in (**i**). **k**, **l** Intensity plot (**k**) and quantification graph (**l**) of integrin α5 in uncontacted and in contacted macrophages. **m** Phase-contrast images of co-cultures after arginine-glycine-aspartic acid (RGD) treatment. **n** Relative proportion of uncontacted/contacted MPs upon RGD treatment (*n* = 10; mean ± SEM). ANOVA, analysis of variance. **o** Immunofluorescence images of wild type (WT) and *ITGA5* knock-out (KO) MPs co-cultured with DPSCs. **p** Relative proportion of uncontacted/contacted MPs (*n* = 10; mean ± SEM). **q** Live imaging showing *ITGA5* KO cells (yellow arrowhead). Scale bar: 100 μm. **r** Time course of the DPSC-MP contact (48 h); frames are colour coded according to the contact status. **s** Quantification of contact duration in WT and KO macrophages (*n* = 5; mean ± SEM).

**Fig. 3:**
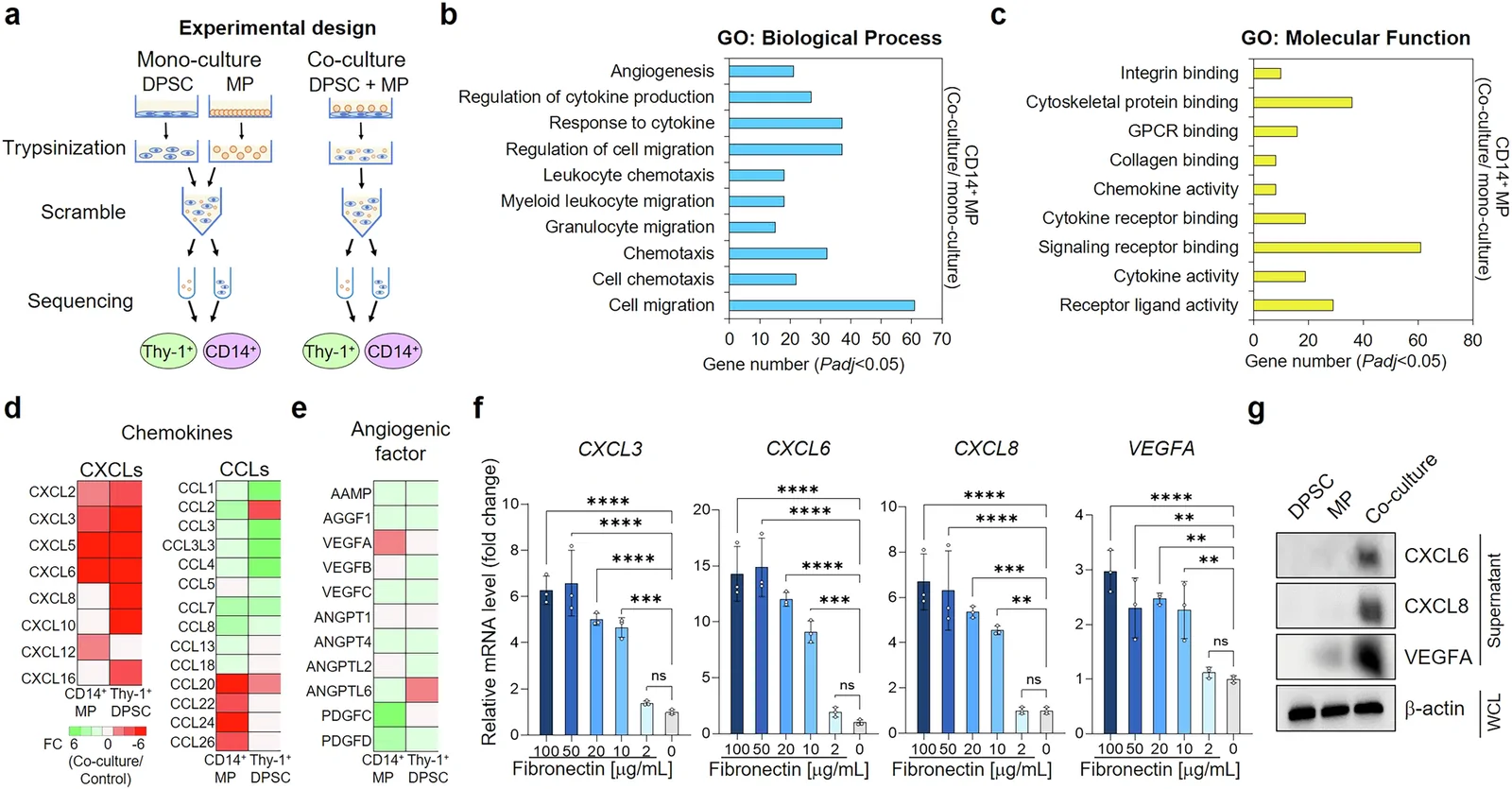
Differential expression of chemokines in the direct co-culture environment. **a** Scheme of the experimental design of bulk RNA sequencing using mono-cultured and co-cultured cells. Thy-1 and CD14 were used as markers of dental pulp stromal cells (DPSCs) and macrophages (MPs), respectively. **b**, **c** Selected Gene Ontology (GO) terms related to cytokines and chemokines in Biological Process and Molecular Function analyses of CD14^+^ MPs (co-culture versus mono-culture). **d** Heatmap of chemokine gene expression in CD14^+^ MPs and Thy-1^+^ DPSCs. **e** Heatmap of angiogenesis-related gene expression in CD14^+^ MPs and Thy-1^+^ DPSCs. **f** mRNA expression levels of pro-angiogenic chemokines (*CXCL3, CXCL6* and *CXCL8*) and *VEGFA* in MPs in response to fibronectin coating in a dose-dependent manner. Analysis of variance, Bonferroni test: ns = non-significant, **P < 0.01, ***P < 0.001, ****P < 0.0001 (*n* = 3; mean ± standard error of the mean (SEM)). **g** Protein expression levels of pro-angiogenic factors under mono-culture and co-culture conditions. Supernatant: cell culture conditioned medium, WCL, whole-cell lysate.

Cells sense and respond to their biomechanical environment through adhesion complexes at both cell–cell and cell–matrix interfaces^32^. Based on this background, two possible mechanisms can be proposed to explain the observed macrophage–DPSC interactions (Fig. 2a). The first involves direct homophilic binding between identical cell-adhesion molecules (CAMs), which facilitates immediate communication between neighbouring cells^33^. The second mechanism occurs through ECM intermediaries, where integrin receptors enable cells to detect and respond to matrix-derived signals, creating cell–ECM–cell communication networks^34^. To identify candidate molecules mediating macrophage–DPSC interactions, we profiled the expression levels of adhesion components in isolated DPSCs and macrophages under co-culture conditions using RNA sequencing (RNA-seq) (Fig. 3a). We first examined the modes of homophilic interactions, focusing on cadherins (*CDH*) and intercellular adhesion molecules (*ICAM, PECAM* and *VCAM)* (Fig. 2b). However, the expression profiles of adhesion molecules in macrophages and DPSCs showed a lack of homophilic matching in the expression patterns, likely due to cell-type discrepancies. We investigated the next hypothetical mechanism involving integrin-mediated cell–ECM interactions (Fig. 2a). DPSCs are recognized as the primary cell type that provides diverse ECM scaffolds in pulp tissues^35^. Our transcriptome profiles revealed that fibronectin (*FN1*) and type I collagen (*COL1A1* and *COL1A2*) were predominantly expressed in DPSCs. To explore the corresponding integrin receptors, we profiled the expression patterns of the integrin family in macrophages derived from co-cultures. *ITGA5* and *ITGB1* were the most abundantly expressed α- and β-subunits, respectively (Fig. 2c). To systematically evaluate the potential ligand–receptor interactions between these cell populations, we applied CellPhoneDB, an algorithm for inferring cell–cell communication, to our RNA-seq dataset^36^. Network visualization revealed that the predominant interactions originated from the FN1 and collagen families (Supplementary Fig. 1a). Moreover, the prediction of ligand–receptor interactions demonstrated multiple statistically significant interactions between DPSCs and macrophages, and the dot plot showed that FN1 interacted with various integrin receptors (ITGA5B1, ITGA4B1 and ITGA3B1) on macrophages (Fig. 2d, e). In addition, the mRNA expression levels of key integrins showed that *ITGA5* and *ITGB1* were predominantly expressed in macrophages (Fig. 2f), which was consistent with the transcriptome profile (Fig. 2c).

Next, we confirmed fibronectin protein expression in human dental pulp tissues. Immunofluorescence analysis revealed that fibronectin was distributed throughout the pulp stroma, with a condensed pattern around the odontoblast layer (Supplementary Fig. 1b). In two-dimensional culture conditions, fibronectin structures were enriched specifically on the dorsal surface of DPSCs, where they formed an organized network that partially colocalized with actin filaments (Fig. 2g). Furthermore, the immunolabelled fibronectin signals showed high proximity to the macrophages (Fig. 2h). Structured illumination microscopy was used to investigate the spatial organization of integrin subunits and fibronectin. Notably, the distribution of integrin α5 in macrophages closely aligned with fibronectin patterns on DPSCs. The clustered integrin α5 was enriched at sites where macrophages and DPSCs were in contact, coinciding with the pattern of fibronectin deposition (Fig. 2i, j). The intensity plot of integrin α5 showed increased signal intensity alongside fibronectin structures with cluster formation, indicating functionally activated forms^37,38^ (Fig. 2k, l). To functionally validate fibronectin-mediated adhesion, we treated cells with a synthetic arginine-glycine-aspartic acid (RGD) peptide that inhibits integrin–ligand interaction^39^. RGD peptide treatment significantly reduced the proportion of macrophage–DPSC interactions in a concentration-dependent manner (Fig. 2m, n). Collagenase treatment had no significant effect on cell–cell interactions (Supplementary Fig. 1c). To investigate the effects of genetic perturbation of the integrin subunit, we used a clustered regularly interspaced short palindromic repeat (CRISPR)-associated protein 9 (Cas9) knockout (KO) system. RAW264.7 cells were transfected with Cas9-RFP alone or co-transfected with Cas9-RFP and single-guide RNA -GFP, and fluorescence-labelled cells were isolated by fluorescence-activated cell sorting (Supplementary Fig. 1d, e). The deletion efficiency of the target sequence was confirmed by quantitative real-time PCR (Supplementary Fig. 1f). Functionally, *ITGA5* KO macrophages presented a significantly reduced proportion of contact with DPSCs compared with wild type cells (Fig. 2o, p). Using live imaging, we found that *ITGA5* KO macrophages showed reduced contact sustainability with DPSC (Fig. 2q). Quantitative results revealed a significant decrease in the duration of the cell-to-cell contact phase (Fig. 2r, s). These findings confirmed that the organization of integrins on macrophages was functionally aligned with fibronectin in DPSCs, thereby regulating intercellular communication. Integrin engagement is known to activate focal adhesion kinase (FAK), leading to its phosphorylation and activation of downstream signalling pathways^40^. To investigate this mechanism, macrophages were cultured on fibronectin-coated surfaces (100 µg/ml), and the expression of phosphorylated FAK (pFAK) was assessed. Notably, pFAK protein levels increased in macrophages cultured on fibronectin-coated surfaces (Supplementary Fig. 1g). We compared pFAK expression between mono-culture and co-culture conditions. The levels of pFAK were significantly higher in the co-culture group than in the mono-culture control group (Supplementary Fig. 1h, i). Taken together, these experimental and theoretical findings suggest that fibronectin–integrin–FAK signalling may serve as an important mediator of macrophage activation.

### Macrophage–dental pulp stromal cell interaction drives a pro-angiogenic chemokine shift

To compare the transcriptomic profiles between the mono- and co-culture conditions, cells from each condition were sorted by fluorescence-activated cell sorting using specific markers: Thy-1 for DPSCs and CD14 for macrophages. The sorted populations were subsequently subjected to bulk RNA-seq (Fig. 3a and Supplementary Fig. 2a). Hierarchical clustering revealed distinct gene-expression patterns in both DPSCs and macrophages under co-culture conditions compared with their mono-culture counterparts (Supplementary Fig. 2b). Differential expression analysis (*P* < 0.05, |log_2_FC | ≥ 2) identified an increase in the number of differentially expressed genes (DEGs), with 586 genes significantly altered, comprising 459 upregulated and 127 downregulated genes in macrophages under co-culture conditions. In DPSCs, 200 genes were upregulated and 249 genes were downregulated (Supplementary Fig. 2c). KEGG pathway analysis based on the DEGs of macrophages predicted multiple enriched pathways associated with various cellular functions. Among these, immune-related pathways, including cytokine and chemokine signalling, showed the most substantial changes (Supplementary Fig. 2d). Additionally, pathways involved in cell survival and adhesion, such as the PI3K–Akt signalling pathway, ECM–receptor interaction, and focal adhesion, were notably enriched (Supplementary Fig. 2d). Gene Ontology (GO) analysis further demonstrated that cytokine- and chemokine-associated terms were significantly enriched in both the Biological Process and the Molecular Function categories (Fig. 3b, c). Therefore, we investigated the expression patterns of cytokine- and chemokine-related DEGs and observed a notable shift in chemokine expression; although most CXC chemokine members were upregulated, the majority of CCL genes were downregulated. In particular, pro-inflammatory chemokines such as *CCL2* (log_2_FC = −2.22) and *CCL3* (log_2_FC = −1.28)^41,42^ were downregulated and various CXC chemokines were upregulated in co-cultured macrophages (Fig. 3d). Among the upregulated gene set, *CXCL2, CXCL3, CXCL5, CXCL6* and *CXCL8* belong to the Glu-Leu-Arg (ELR)-positive (ELR^+^) subgroup, characterized by a conserved ELR motif at their N-terminus, which is essential for their angiogenic activity^43^. These observations suggested a potential role for CXC chemokines in promoting angiogenesis through interactions between macrophages and DPSCs (Fig. 3d). Additionally, gene-expression profiling of angiogenic markers revealed a significant increase in *VEGFA* expression in the co-culture group (Fig. 3e). Notably, the GO terms, “angiogenesis” (GO:0001525), “sprouting angiogenesis” (GO:0002040) and “cell migration involved in sprouting angiogenesis” (GO:0002042) were also identified as significantly enriched Biological Process pathways under co-culture conditions (Fig. 3b). Notably, a similar chemokine expression pattern was observed in DPSCs, which was likely the result of reciprocal interactions with macrophages mediated by a shared ECM environment (Fig. 3d, e). To determine whether the observed chemokine-switching effects were mediated by fibronectin, macrophages were seeded on plates coated with increasing concentrations of fibronectin and the mRNA expression levels of selected pro-angiogenic factors (*CXCL3, CXCL6, CXCL8* and *VEGFA*) were quantified. We observed a dose-dependent upregulation of these pro-angiogenic factors with increasing fibronectin concentrations (Fig. 3f). Finally, the protein levels of CXCL6, CXCL8 and VEGF-A in the culture supernatant were significantly elevated under co-culture conditions compared with those in the mono-culture, confirming the enhanced secretion of angiogenic factors (Fig. 3g). To investigate the underlying mechanism, macrophages were treated with RGD peptides to disrupt integrin–ligand interactions. Under these conditions, RGD treatment significantly reduced the mRNA expression levels of *CXCL2/3/6/8* and *VEGFA* compared with those in the control condition (Supplementary Fig. 3a). Consistent with the transcription results, the protein levels of CXCL6, CXCL8 and VEGFA in the culture supernatants were significantly lower in the RGD-treated group than in the control group (Supplementary Fig. 3b). Next, we performed an small interfering RNA-mediated knockdown of *FN1* in DPSCs (Supplementary Fig. 3c). In an in vitro co-culture system, the protein expression levels of CXCL6, CXCL8 and VEGFA were downregulated in response to *FN1*-silenced DPSCs (Supplementary Fig. 3d). We further characterized macrophage phenotypic polarization by analysing the established markers of M1 and M2 macrophages under mono- and co-culture conditions. CD86 is widely recognized as a marker of pro-inflammatory M1 macrophages, whereas CD206 is a representative marker of anti-inflammatory and pro-angiogenic M2 macrophages^44^. Notably, CD206 expression markedly increased in the co-culture group compared with that in the mono-culture control (Supplementary Fig. 4a, b). In addition, flow cytometry revealed that the proportion of CD206^+^ macrophages increased under co-culture conditions, whereas the CD86^+^ population did not show a significant change (Supplementary Fig. 4c–g). Collectively, these results indicated that macrophages underwent a phenotypic shift towards M2-like macrophages upon interaction with DPSCs. Taken together with our transcriptomic data, these findings suggest that co-culture conditions induce the upregulation of specific chemokine genes, reflecting a shift towards a pro-angiogenic transcriptional programme. Chemokine-mediated signalling reprogramming may also contribute to the suppression of inflammatory responses, thereby supporting a regeneration-prone microenvironment.

### Deciphering cell-to-cell communications in dental pulp

**Fig. 4:**
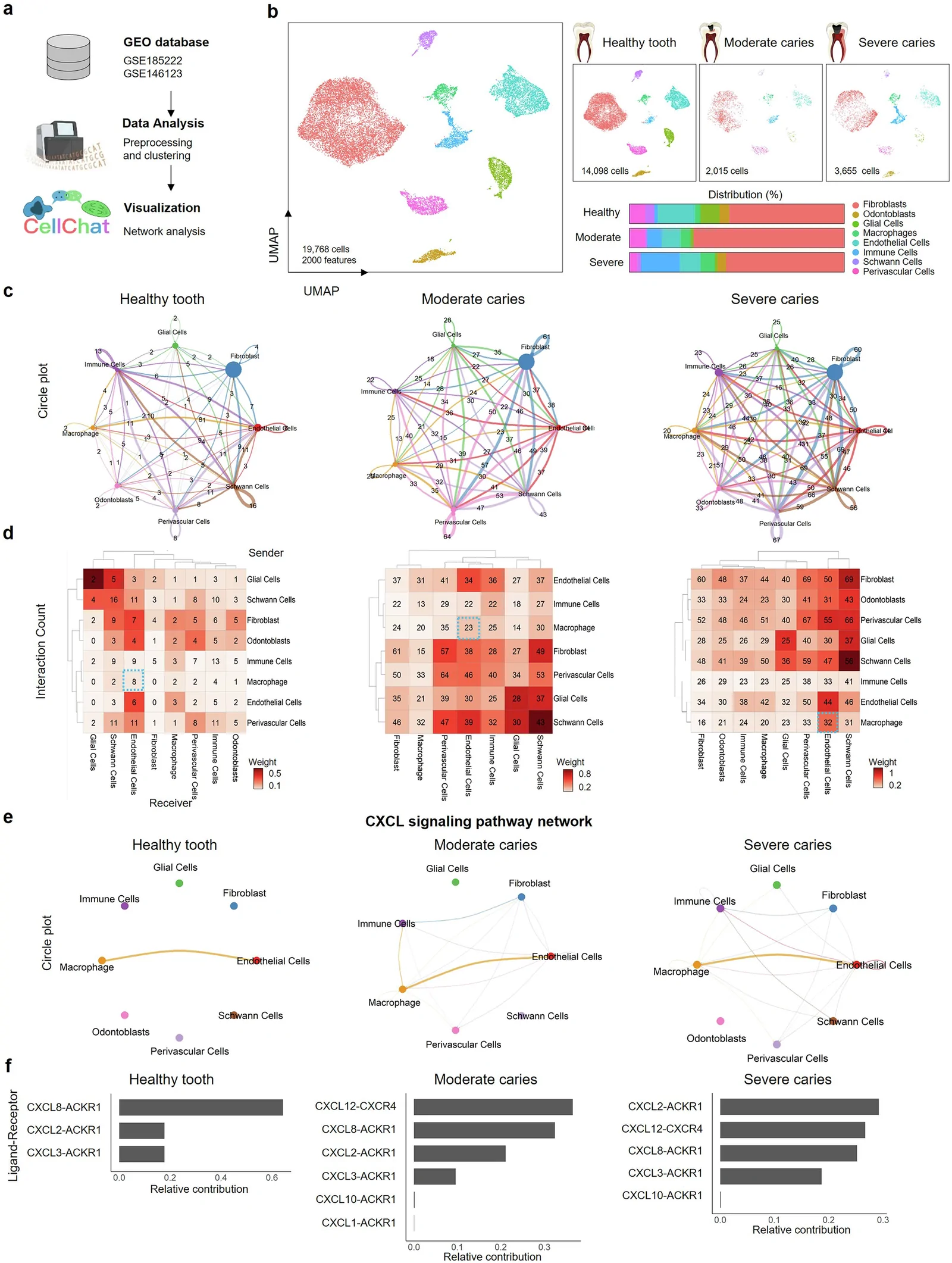
Deciphering macrophage interactions in the dental pulp. **a** Schematic workflow illustrating the construction and analysis pipeline of the human dental pulp single-cell atlas. **b** (Left) Uniform manifold approximation and projection (UMAP) visualization of 19,768 single cells derived from dental pulp tissue. (Right, *Upper*) UMAP proportion plots, separated by total group: healthy (14,098 cells), moderate (2,015 cells), and severe (3,655 cells) populations, respectively (*n* = 2, *n* = 1 and *n* = 1, respectively). (Right, *Lower*) Cellular distribution profiles across healthy teeth, moderate caries and severe caries groups. **c** Global cell–cell communication networks between different cell subsets. The line width represents interaction strength. Different colours in the circle plot represent different cell groups. **d** Heatmaps showing the number and weight of interactions of different cell subsets. **e** Inferred CXCL signalling networks among cell subsets. Line width corresponds to the predicted communication probability. **f** Relative contribution of each ligand–receptor pair to the CXCL signalling, with values normalized to a total sum of 1.

The dental pulp exhibits extensive cellular crosstalk among diverse cell populations. In addition to macrophages and DPSCs, it consists of a wide variety of cell types, reflecting its remarkable cellular heterogeneity and complexity^45^. To investigate communication dynamics during caries progression, we integrated previously reported single-cell RNA-seq databases (GSE185222 and GSE146123)^45,46^, including single-cell transcriptomic profiles of pulp tissues from healthy teeth (14,098 cells), teeth with moderate caries (2,015 cells) and teeth with severe caries (3,655 cells) (Fig. 4a). Uniform manifold approximation and projection (UMAP) revealed eight distinct cell populations in dental pulp: fibroblasts, odontoblasts, glial cells, macrophages, endothelial cells, immune cells, Schwann cells and perivascular cells (Fig. 4b). Marker gene profiles were used to validate the identity of each cluster (Supplementary Fig. 5a, b). The composition of the major clusters showed that endothelial cells decreased from 16.8% in healthy pulp to 8.6% in moderate caries and 9.5% in severe caries, whereas perivascular cells decreased from 7.9% to 7.4% and 3.7%, respectively. Conversely, inflammatory cells markedly expanded, with immune cells increasing from 1.5% in healthy pulp to 6.7% in moderate lesions and 18.1% in severe lesions. Macrophages consistently increased, representing 2.6%, 4.7%, and 6.8% under healthy, moderate, and severe conditions, respectively (Supplementary Fig. 5c). Next, the CellChat computational framework was applied to the single-cell (sc) RNA-Seq dataset. This approach enables quantitative inference and analysis of intercellular communication by mapping ligand–receptor interactions and interpreting signalling networks^47^. The circular plot reveals an increased number and strength of interactions in the severe caries group, particularly among macrophages, endothelial cells, fibroblasts and other immune cells, suggesting a more complex and reciprocal microenvironment under pathological conditions (Fig. 4c). The total interaction counts and signalling strengths progressively increased from the healthy group to the severe caries group (interaction count: 278 (healthy), 1,646 (moderate) and 2,470 (severe); signaling strength: 7.17 (healthy), 14.23 (moderate) and 21.41 (severe)) (Supplementary Fig. 5d). The heatmap aggregated from all signalling pathways showed a marked increase in both the number and strength of intercellular communications in moderate and severe caries compared with those in healthy teeth (Fig. 4d). Notably, the number of interactions between macrophages and endothelial cells is substantially increased in severe caries. This suggests that macrophages actively engage with the vascular compartment, potentially contributing to the key intercellular interactions within the inflamed pulp microenvironment. Specifically, the CXCL signalling pathway, marked by the activation of inflammatory and chemokine-mediated pathways, underscored the strengthened interactions between macrophages (as senders) and both endothelial and immune cells (as receivers) in carious pulp tissues (Fig. 4e). Furthermore, multiple CXCL receptor pairs were upregulated during disease progression (Fig. 4f). In addition, centrality measures identified key mediating cell types, particularly macrophages and endothelial cells, in both CXCL and VEGF pathways (Supplementary Fig. 6a–c). Notably, these findings are highly consistent with the bulk RNA-seq results (Fig. 3), supporting the critical role of macrophages in angiogenesis in damaged pulp tissues. In addition, the FN1 signalling pathway revealed that fibroblasts serve as the primary senders of FN1 signalling within the pulp microenvironment, indicating that fibroblasts are the primary cellular source of fibronectin (Supplementary Fig. 6d, e).

### Validation of pro-angiogenic effect via fibronectin-mediated immune–stromal interaction

**Fig. 5:**
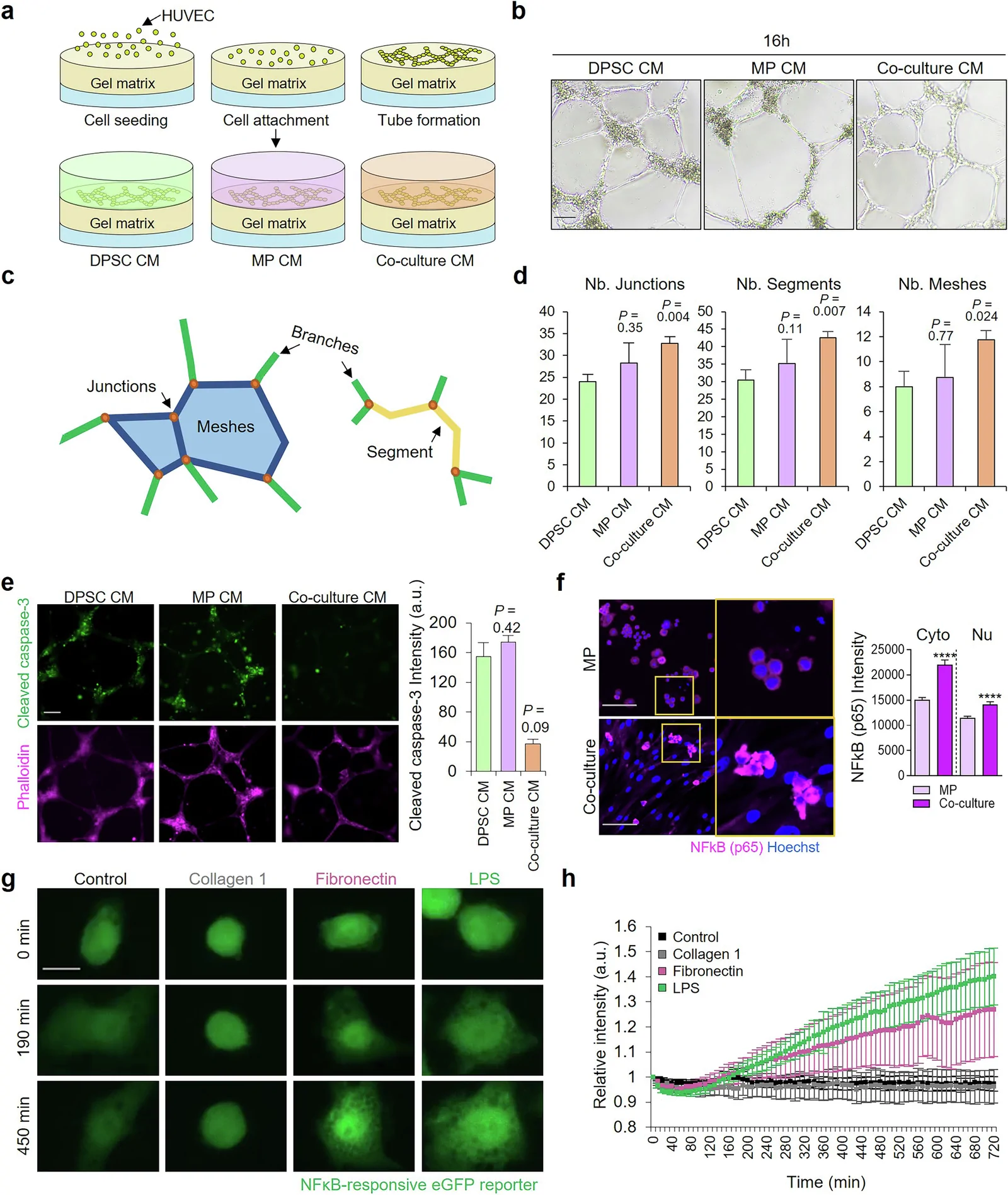
Fibronectin-mediated crosstalk promoting a pro-angiogenic secretome. **a** Scheme of the experimental design for the tube formation assay. **b** Microscopic images of tube formation after 16 h of treatment with conditioned medium (CM). Scale bar: 100 μm. **c** Diagram illustrating the quantification parameters for angiogenesis. **d** Quantitative analysis of angiogenic parameters using the Angiogenesis Analyzer in each CM condition. DPSC, dental pulp stromal cell; MP, macrophage; Nb. Junctions, number of junctions; Nb. Mesh, number of meshes; Nb. Segments, number of segments. The analysis of variance test was also performed. **e** Fluorescence images of the apoptotic marker cleaved caspase-3 and phalloidin in each CM condition, along with quantification of cleaved caspase-3 intensity. Scale bar: 100 μm. Student’s *t*-test was also performed. a.u.: arbitrary units. **f** Immunofluorescence of NFκB (p65) in MP mono- and co-cultures, with quantification of cytoplasmic (Cyto) and nuclear (Nu) expression. Scale bar: 100 μm. Student’s *t-*test; ****P < 0.0001. **g**, **h** Live-cell imaging of macrophages transfected with an NFκB-responsive eGFP reporter cultured on fibronectin, and quantification of eGFP signal intensity. Collagen 1 coating was used as a negative control, and LPS (100 ng/ml) treatment was used as a positive control. Scale bar: 10 μm. a.u.: arbitrary units.

Based on previous findings, we next evaluated the pro-angiogenic potential of the co-culture environment using human umbilical vein endothelial cells (HUVECs). A tube formation assay was performed by culturing HUVECs on Matrigel-coated plates and treating them with conditioned medium (CM) collected under mono-culture or co-culture conditions (Fig. 5a). Notably, HUVECs treated with co-culture CM exhibited enhanced tube formation capacity compared with those treated with mono-culture (DPSC or macrophage) CM (Fig. 5b). The co-culture CM-treated group showed significantly enhanced key angiogenic parameters, including the number of junctions, segments and meshes, relative to the mono-culture groups (Fig. 5c, d). Notably, we observed the formation of membrane blebs, a characteristic morphological feature of apoptosis^48^, in endothelial cells treated with the mono-culture CM. Such apoptotic blebbing was notably absent in endothelial cells exposed to co-cultured CM. To validate this observation, we performed immunostaining for cleaved-caspase-3 and demonstrated reduced cleaved-caspase-3 levels in endothelial cells exposed to co-cultured CM (Fig. 5e). These results are consistent with those of previous studies reporting that pro-angiogenic chemokines and VEGF promote endothelial survival^43,49,50^. Thus, the altered secretome under co-culture conditions may contribute to improved endothelial survival and angiogenesis through these key mediators.

To further determine whether integrin–ligand interactions are required for the observed pro-angiogenic and survival effects, we disrupted these interactions using an RGD peptide. Specifically, macrophages were primed with the RGD peptide prior to co-culture with DPSCs, and the outcomes were compared with those of the untreated co-culture group. Tube formation was assessed in HUVECs, and quantitative analysis revealed that RGD peptide treatment significantly reduced the number of junctions, segments and meshes, indicating impaired angiogenic network formation (Supplementary Fig. 7a). Consistently, cell-survival analysis based on cleaved caspase-3 immunostaining demonstrated a significant increase in apoptotic cells in the RGD-treated group compared with the co-culture group (Supplementary Fig. 7b). These results indicate that integrin–ligand interactions are functionally required for the pro-angiogenic and survival-promoting effects observed in the co-culture system. To further investigate the contribution of downstream pro-angiogenic mediators, we performed pathway-inhibition experiments using CXCR2 (SB225002) and VEGFR (AAL-993) inhibitors in conditioned media derived from a co-culture system. CXCR2 signalling, which mediates the activity of CXCL3, CXCL6 and CXCL8, was inhibited by SB225002, whereas VEGFR signalling, the primary receptor pathway for VEGFA, was blocked by AAL-993^51^. Functional angiogenic capacity was evaluated using an HUVEC tube formation assay. Both inhibitors led to an overall reduction in tube formation. Quantitative analysis showed that the number of segments significantly decreased in both inhibitor-treated groups. Additionally, the number of junctions and meshes was significantly reduced in the VEGFR inhibitor-treated group. Although these parameters were not statistically significant in the CXCR2 inhibitor group, a similar decreasing trend was observed (Supplementary Fig. 7c). Cell survival was assessed by immunostaining for cleaved caspase-3. Both inhibitor treatments resulted in a significant increase in the number of apoptotic cells, indicating the reduced survival of HUVECs (Supplementary Fig. 7d). Collectively, these results demonstrate that inhibition of CXCR2- and VEGFR-mediated signalling attenuates angiogenic activity and cell survival, supporting the contribution of these downstream pro-angiogenic pathways in mediating the observed effects.

To investigate the underlying transcriptional regulators mediating this response, we focused on NFκB because a previous study reported that integrin-specific binding to fibronectin activates NFκB-dependent gene expression^52^. To test this hypothesis, we performed immunostaining for NFκB (p65) to assess transcription factor activity in macrophages co-cultured with DPSCs. The intensity of NFκB (p65) was markedly increased in both the cytoplasm (146.2% ± 17.7%) and the nucleus (123% ± 20.5%) of macrophages under co-culture conditions, indicating activation of NFκB signalling (Fig. 5f, g). To determine whether this activation was mediated by fibronectin, we employed an NFκB reporter system^53^ and conducted live-cell imaging to monitor NFκB activity in real time from the initial point of cell attachment. The GFP-based reporter signals increased significantly over time in the LPS-treated group, validating the reporter system. Notably, cells seeded on fibronectin-coated surfaces exhibited an approximately 90.7% increase in GFP intensity compared with the LPS-treated group, with the signal continuing to increase over a 12-h period. In contrast, the cells cultured on collagen 1 showed no changes in signal activation (Fig. 5g, h). Together, these findings indicate that fibronectin acts as a critical mediator of the interaction between DPSCs and macrophages in a co-culture environment, facilitating NFκB activation in macrophages. This immune–stromal interaction results in a pro-regenerative secretome enriched in pro-angiogenic mediators, thereby enhancing endothelial-cell survival and promoting vascular formation.

### Effect of fibronectin-enhanced revascularization on pulp-dentin-like tissue formation and continued root development in vivo

**Fig. 6:**
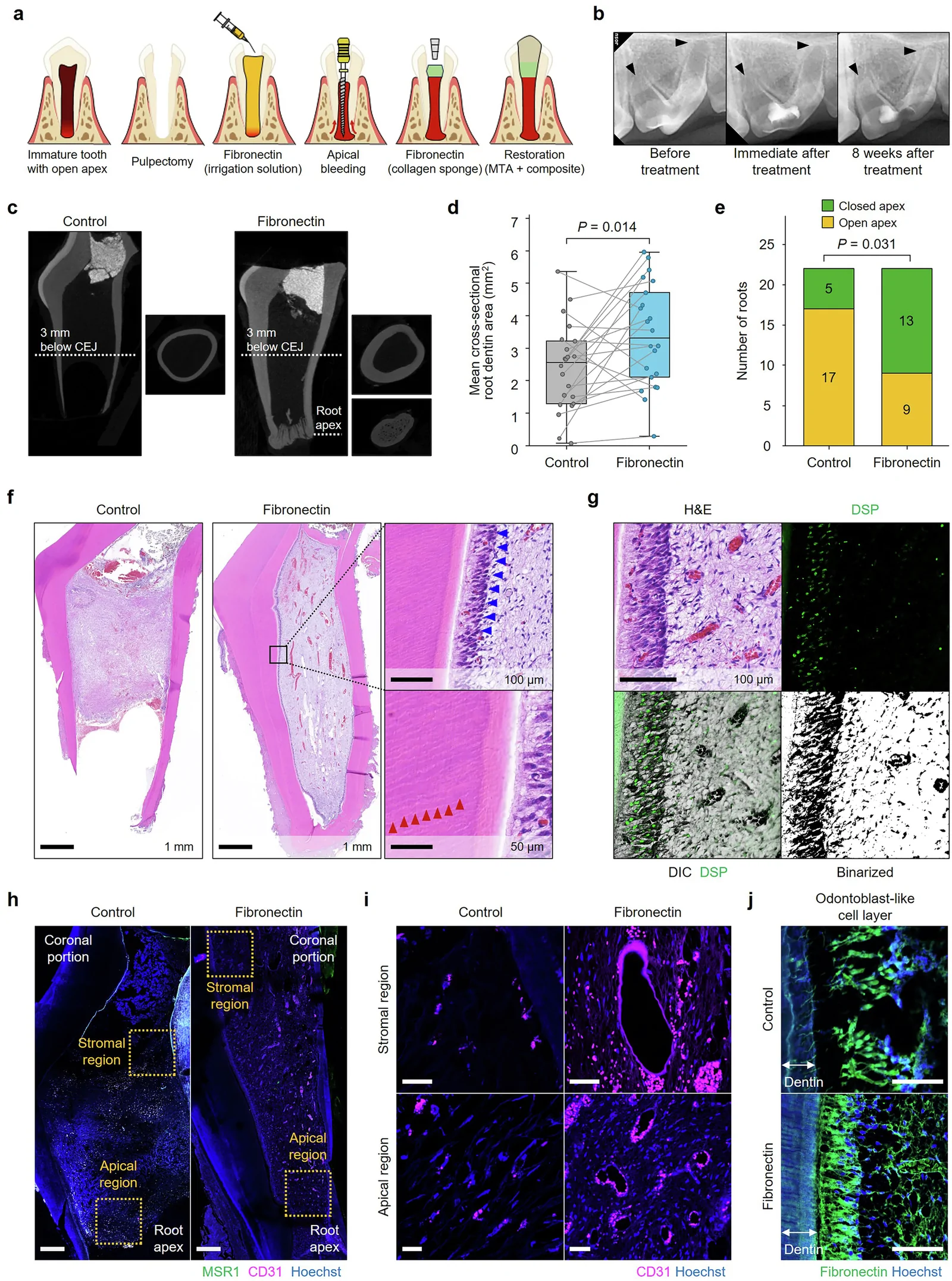
Fibronectin-promoted dental pulp revascularization in immature canine teeth. **a** Revascularization scheme. In the control group, a PBS-soaked collagen sponge was placed in direct contact with the induced blood clot. In the fibronectin-treated group, fibronectin was applied by final canal irrigation, and an fibronectin-soaked collagen sponge of the same size was placed in the same position. **b** Representative periapical radiographs taken before, immediately after and 8 weeks after revascularization. Black arrowheads indicate root-wall thickening and apical closure. **c** Micro-CT (μCT) images of control and fibronectin-treated teeth. Cross-sectional images were obtained 3 mm below the cementoenamel junction (CEJ). The apical image of the fibronectin-treated root showed apical closure with multiple channel-like structures at the root apex, consistent with the apical architecture of mature canine teeth. **d** Quantitative μCT analysis of mean cross-sectional root dentin area in control and fibronectin-treated roots (*n* = 22 roots per group from three independent canine animals). Each dot represents one root, and values from paired roots are connected by lines. Box plots show the median and interquartile range. Statistical significance was determined by a paired *t-*test. **e** Quantification of apical closure status in control and fibronectin-treated roots based on μCT analysis (*n* = 22 roots per group from three independent canine animals). Numbers in bars indicate the number of roots in each category. Statistical significance was determined by two-sided Fisher’s exact test. **f** Haematoxylin and eosin (H&E) staining of longitudinal tooth sections showing newly formed hard-tissue and pulp-like tissue organization. In fibronectin-treated roots, blue arrowheads indicate an organized layer of polarized odontoblast-like cells, and red arrowheads indicate dentinal tubule-like structures. Scale bars: as indicated. **g** Immunostaining for dentin sialoprotein (DSP) in the odontoblast-like cell layer of fibronectin-treated roots. DSP-positive staining along the odontoblast-like cell layer supports odontoblastic differentiation within the regenerated pulp-like tissue. Scale bar: 100 μm. DIC differential interference contrast. **h** Immunostaining of tooth sections from control and fibronectin-treated groups. Scale bars: 500 μm. **i** Higher-magnification images of boxed areas in **h** showing vascular organization in the stromal and apical regions. Scale bars: 50 μm. **j** Fibronectin immunostaining showing enhanced fibronectin localization in the extracellular matrix adjacent to the organized odontoblast-like cell layer. Scale bars: 100 μm.

Finally, to validate our in vitro findings and assess the translational potential of fibronectin-mediated interactions, we established a preclinical pulp revascularization model using immature premolar teeth in canines (Fig. 6a). The in vivo analysis included three independent canines. Within these animals, root-level outcomes were assessed in 22 roots per group using a split-mouth design to compare control and fibronectin-treated roots. Both groups received the same collagen sponge scaffold, whereas the fibronectin-treated group received irrigation with fibronectin solution and an fibronectin-soaked collagen sponge.

Periapical radiographs and μCT analyses were performed 8 weeks after revascularization (Fig. 6b, c). Quantitative μCT analysis showed that the mean cross-sectional root dentin area was significantly greater in fibronectin-treated roots than in control roots (3.40 ± 1.58 vs 2.39 ± 1.34 mm²; paired *t-*test, *P* = 0.014; Fig. 6d). μCT analysis further showed that apical closure was more frequently observed in fibronectin-treated roots (13/22 roots, 59.1%) than in control roots (5/22 roots, 22.7%) (Fisher’s exact test, *P* = 0.031; Fig. 6e). These findings indicate that fibronectin treatment promotes continued root development and apical maturation in a canine revascularization model. The histological examination further supported these findings (Fig. 6f, g, and Supplementary Fig. 8). H&E staining showed that the fibronectin-treated roots exhibited newly formed hard-tissue with an organized layer of polarized odontoblast-like cells and dentinal tubule-like structures (Fig. 6f). Immunohistochemical staining for dentin sialoprotein (DSP), an odontoblast/dentin matrix-associated marker, showed DSP-positive staining along the odontoblast-like cell layer in the fibronectin-treated roots (Fig. 6g), supporting odontoblastic differentiation within the newly formed pulp–dentin-like tissue. Additional histological comparisons showed that the fibronectin-treated roots contained more organized pulp–dentin-like tissue, including abundant blood vessels, dentin-like tissue formation, an odontoblast-like cell layer, and apical hard-tissue closure, with multiple channel-like structures resembling the apical architecture of mature canine teeth^54^ (Supplementary Fig. 8a–d). In contrast, the control roots showed loosely organized amorphous fibrous tissue within the canal space, fewer blood vessels, thin root dentin walls, no evident tertiary dentin-like deposition and incomplete apical closure (Supplementary Fig. 8e–h).

Immunofluorescence analyses further demonstrated enhanced vascularization in the fibronectin-treated group, with prominent vascular-marker-positive structures observed in both the apical and stromal regions (Fig. 6h, i). Additionally, distinct fibronectin localization was detected along the regenerated odontoblast layer, suggesting the establishment of an organized ECM microenvironment within the regenerated tissue (Fig. 6j). Compared with the control group, the fibronectin-treated group exhibited more abundant CD14^+^ macrophages in both odontoblastic cell layers (Supplementary Fig. 9a) and the stromal region (Supplementary Fig. 9b). Notably, CD14^+^CD163^+^ M2 macrophages were predominantly localized to the stromal region of the fibronectin-treated group, where their percentage was significantly increased compared with the control group (Supplementary Fig. 9b, c).

Together, these in vivo findings suggest that fibronectin application during revascularization enhances root dentin formation and apical maturation, and promotes the formation of an organized pulp–dentin-like tissue structure. These results support the hypothesis that fibronectin contributes to a pro-regenerative microenvironment associated with immune–stromal crosstalk and angiogenic signalling during dental pulp regeneration, which is consistent with the enhanced tissue organization, vascularization and root maturation observed in the experimental group.

## Discussion

This study demonstrates that the engagement of fibronectin with integrin receptors on macrophages plays a pivotal role in modulating the dental pulp microenvironment during the transition from tissue damage to regeneration. Using a combination of spatial-tissue analysis and co-culture systems, we revealed that macrophages are dynamically redistributed within the infected dentin–pulp interface and establish functional contact with DPSCs via fibronectin matrices. This fibronectin–integrin interaction not only reorganizes the cytoskeletal architecture of macrophages but also activates NFκB signalling, thereby driving a shift towards a pro-angiogenic transcriptional programme marked by elevated pro-angiogenic CXCL chemokines and VEGFA expression. These changes significantly enhanced the angiogenic potential of the pulp tissue. Finally, we demonstrated the regenerative potential of this immune–stromal axis in a preclinical canine pulp revascularization model, in which fibronectin treatment enhanced vascularization, root-dentin formation, apical maturation, and organized pulp–dentin-like tissue formation (Supplementary Fig. 10).

Fibronectin is a well-established major structural constituent of the ECM and is a central signalling platform that orchestrates intracellular pathways through integrin engagement, called outside–in signalling^55^. In addition to serving as a physical scaffold, fibronectin dynamically modulates cell adhesion, migration and survival through biochemical and mechanical cues. Fibronectin has also been employed in scaffold-based therapeutic strategies, and its critical role in tissue regeneration is well documented. For example, fibronectin nanofibres promote wound closure and skin repair^56^. Moreover, exogenous fibronectin treatment restores muscle stem-cell function and enhances muscle regeneration in aged tissues^57^. Previous studies have reported the regenerative function of fibronectin as a direct structural effector in stromal cells. However, our findings suggest a novel functional link between fibronectin and the angiogenic process, with fibronectin acting as a mediator of immune–stromal cell interactions. This fibronectin-mediated crosstalk may exert multidisciplinary effects on tissue regeneration by connecting multiple cell types responsive to fibronectin networks. Potential links among these diverse cell types were demonstrated by our integrated single-cell analysis (Fig. 4e), and similar interactive patterns were also observed in nerve regeneration^58^. In this process, macrophages may serve as key transducers of ECM-mediated signalling to neighbouring cells, accompanied by shifts in the microenvironment towards the remodelling phase.

Dental pulp is composed of complex cell populations with low cell densities, and the surrounding mineralized tissues make it particularly challenging to investigate cell-to-cell interactions. To overcome this limitation, we used scRNA-seq, a powerful technique that preserves cellular information with high resolution and is particularly useful for analysing tissues with high heterogeneity. By integrating scRNA-seq data with the CellChat algorithm, we successfully dissected cell–cell communication in pulp tissue according to the different phases of caries progression. The most prominently enriched ligand–receptor interactions involved the CXCL family chemokines and their associated receptors (Fig. 4f), consistent with the bulk RNA-seq results from in vitro co-culture experiments (Fig. 3d). Interestingly, ligand–receptor prediction analysis revealed the enrichment of non-canonical atypical chemokine receptor 1 (ACKR1) in infected pulp tissues (Fig. 4f). ACKR1 is primarily expressed in endothelial cells and plays a crucial role in the regulation of chemokine gradients and immune-cell positioning^59^. In contrast to previously known functions, the interaction between ACKR1-expressing endothelial cells and macrophages via chemokine signalling may represent a novel axis involved in the regenerative response during pulpal pathology. In addition, circle plots illustrating multicellular interactions showed markedly enhanced connectivity of Schwann, endothelial and perivascular cells with various other cell types as the caries progressed (Fig. 4c). These findings underscore the potentially critical role of neurovascular components in the response to pulp-tissue injury and offer new insights into the mechanisms that may support pulp–dentin-like tissue repair and regeneration. Therefore, understanding intercellular communication is essential to elucidate the expanding roles and functional plasticity of diverse cell types in the regenerative process.

Pulp revascularization relies on the regenerative potential of apical and periodontal stromal cells to differentiate and form a richly vascularized connective tissue-filled structure^60^. Angiogenesis is a central component of this regenerative process and is essential not only for tissue revitalization but also for sustained pulp^61^. In our preclinical canine revascularization model, fibronectin treatment increased root dentin formation and the frequency of apical closure in addition to enhancing neovascularization. We propose that the potent regenerative effects induced by fibronectin are likely the result of multiple biological mechanisms. During pulp angiogenesis, various factors, such as vascular endothelial growth factor (VEGF), platelet-derived growth factor (PDGF-AB), placental growth factor (PlGF) and angiopoietins, are delivered through newly formed blood vessels. These vessels also recruit mesenchymal stromal cells, contributing to continued root development and periapical healing^60,62^. Additionally, fibronectin treatment directly promotes MSC differentiation, as evidenced by the upregulation of dentin sialophosphoprotein (DSPP) expression in DPSCs^63^. Another noteworthy observation from our preclinical model was the localization of fibronectin adjacent to the organized odontoblast-like cell layer (Fig. 6j), which closely resembled the intact odontoblast layer observed in human teeth (Supplementary Fig. 1b). This well-organized cellular pattern suggests that fibronectin may not function solely as a passive scaffold but may actively contribute to the organization of endogenous pulp cells within the regenerated tissue. The distinct localization of fibronectin along the regenerated odontoblast layer further supports its role in establishing a regenerative microenvironment. Consistent with this interpretation, fibronectin may reinforce the ECM and stabilize the local microenvironment, both of which are essential for the formation and maintenance of the odontoblast layer^64^.

The therapeutic properties of fibronectin demonstrated in this study provide significant opportunities for advancing dental treatment. For example, immature teeth with necrotic pulps represent a significant clinical challenge because conventional root canal treatment results in arrested root development and increased fracture susceptibility^65^. Our demonstration that fibronectin application enhances root development, apical maturation and organized pulp–dentin-like tissue formation suggests a potential direction for regenerative approaches in dentistry. Moreover, given that fibronectin is an endogenous protein with established biocompatibility, its clinical implementation is expected to carry a minimal risk of adverse reactions, making it an attractive therapeutic candidate compared with exogenous growth factors or synthetic biomaterials. Furthermore, the integration of fibronectin-based strategies may be extended to broader applications in regenerative medicine. The angiogenic capabilities of fibronectin suggest its potential utility in soft-tissue regeneration, bone-tissue engineering and other scaffold-based therapeutic interventions. These cross-disciplinary applications underscore the translational potential of our findings beyond dentistry.

Although these findings are promising, their translation from a canine revascularization model to human clinical practice requires careful validation because species-specific differences in pulp biology and regenerative capacity may exist. Further investigations are needed to optimize fibronectin-delivery parameters, including concentration, application duration and carrier material selection. Long-term follow-up studies are essential to assess the stability, functionality and biological integration of regenerated tissues over extended periods. Future research should explore the potential synergistic effects of fibronectin with other bioactive molecules, such as growth factors, which may further enhance regenerative outcomes. Ultimately, well-designed clinical studies are necessary to validate these preclinical findings and establish standardized treatment protocols for fibronectin-enhanced regenerative treatment procedures for the clinical implementation of this promising therapeutic approach.

### Supplementary information

Supplementary information accompanies the manuscript on the *Experimental & Molecular Medicine* website (http://www.nature.com/emm/).

## Supplementary information

Supplementary Information

bulk RNA-seq data

## Data Availability

All data needed to evaluate the conclusions in the paper are present in the paper and/or the supplementary information files. Our data have been deposited in the Gene Expression Omnibus (GEO) under accession number GSE299319. Additional data related to this paper may be requested from the authors.
