# Supplementary material for: Fibronectin-guided immune–stromal cell crosstalk promotes angiogenesis in pulp–dentin complex regeneration
Source: Exp Mol Med. 2026 Oct 6;58(10):3499–513. doi: 10.1038/s12276-026-01863-4 (PMC13737076; doi:10.1038/s12276-026-01863-4)
Supplement: Supplementary file 1 [file 12276_2026_1863_MOESM1_ESM.docx]

Supplementary information for

**Fibronectin-guided immune–stromal cell crosstalk promotes angiogenesis in pulp–dentin complex regeneration**

**Authors:** Sol Jeong^1†^, So Young Park^1†^, Hyemin Ku^2^, Byeong Soo Eom^1^, Dohyun Kim^2*^, Jin Man Kim^1,3,4*^

**Affiliations:**

^1^Department of Oral Microbiology and Immunology, School of Dentistry and Dental Research Institute, Seoul National University; Seoul, 08826, Republic of Korea

^2^Department of Conservative Dentistry and Oral Science Research Center, Yonsei University College of Dentistry; 50-1 Yonsei-ro, Seodaemun-gu, Seoul 03722, Republic of Korea

^3^Dental Multiomics Center, School of Dentistry and Dental Research Institute, Seoul National University; Gwanak-ro 1, Gwanak-gu, Seoul 08826, Republic of Korea

^4^Innovative Pharmaceutical Sciences Program, College of Transdisciplinary Innovations, Seoul National University; Gwanak-ro 1, Gwanak-gu, Seoul 08826, Republic of Korea

*To whom correspondence: [jinmankim@snu.ac.kr](mailto:jinmankim@snu.ac.kr); [dohyun0kim@yuhs.ac](mailto:dohyun0kim@yuhs.ac)

^†^These authors contributed equally to this work.

Sol Jeong *et al.*

**This file includes:**

Figs. S1 to S10

Tables S1 to S2

Materials and Methods

**Supplemental Figures**

**Fig. S1. Validation of signaling activity in macrophage–DPSC interactions.** (**a**) Sankey diagram illustrating key ligand-receptor connections from the sender cell (DPSC) to the receiver cell (MP). (**b**) Immunofluorescence staining of normal human teeth showing fibronectin (green). DAPI (blue) was used as a counterstain for nuclei. Scale bar: 50 μm. (**c**) Phase contrast images of direct co-cultures in control and collagenase-treated groups. Scale bar: 200 μm. (**d**) Flow cytometry analysis of Cas9 and Cas9^+^sgRNA in transfected RAW264.7 cells. Boxed areas indicate RFP-positive and GFP-positive populations. (**e**) Fluorescence images of sorted cells showing Cas9 (magenta) and sgRNA (green). Scale bars: 200 μm. (**f**) Relative mRNA expression levels of *ITGA5* of control, Cas9 and *ITGA5* KO (n = 3; mean ± SEM). (**g**) Protein expression levels of phosphorylated FAK (pFAK, *upper*) and total FAK (*middle*) upon coating with 100 μg/mL fibronectin. β-actin (*lower*) was used as a loading control. (**h**) Immunofluorescence staining of phosphorylated FAK (pFAK, green) in MPs under mono-culture and co-culture conditions. Nuclei were stained with Hoechst (blue). Merged confocal fluorescence and phase-contrast microscopy images. Scale bars: 50 μm. (**i**) Quantification of pFAK fluorescence intensity in MPs under mono-culture and co-culture conditions (n = 10; mean ± SEM).

**Fig. S2. Alteration of cytokine and chemokine profiles in direct co-culture conditions.** (**a**) Flow cytometry sorting of cell populations under mono-culture and co-culture conditions. Thy-1 was used as a marker for dental pulp stromal cells (DPSCs), and CD14 was used as a marker for macrophages (MPs). (**b**) Hierarchical clustering heatmap of CD14⁺ MPs and Thy-1⁺ DPSCs based on genes with statistically significant differences (adjusted *P* < 0.05) between mono-culture and co-culture conditions. (**c**) Differentially expressed gene counts in CD14⁺ MPs and Thy-1⁺ DPSCs (co-culture vs. mono-culture; adjusted *P* < 0.05, |log₂FC| ≥ 2). (**d**) KEGG pathway analysis showing the top 20 enriched and categorized pathways in each cell type (co-culture vs. mono-culture).

**Fig. S3. Inhibition of the FN–integrin axis suppresses pro-angiogenic chemokine secretion.** (**a**) mRNA expression levels of *CXCL2, CXCL3, CXCL5, CXCL6, CXCL8,* and *VEGFA* in macrophages in response to increasing concentrations of RGD peptide. ANOVA followed by Bonferroni test: ns = non-significant, **P* < 0.05, ***P* < 0.01, ****P* < 0.001, *****P* < 0.0001 (n = 3; mean ± SEM). (**b**) Protein expression levels of pro-angiogenic factors in macrophages treated with RGD peptides in mono-culture and co-culture conditions. (**c**) Relative mRNA expression of siRNA-mediated knockdown of *FN1* at 48 h (n = 3; mean ± SEM). (**d**) Protein expression levels of pro-angiogenic factors in *FN1*-silenced DPSCs. Supernatant: cell culture-conditioned medium; WCL: whole cell lysate.

**Fig. S4. M2 polarization in DPSC–macrophage co-culture conditions.** (**a**) Representative immunofluorescence images showing CD86 (red), CD206 (green), and nuclei (Hoechst, blue). Scale bar: 50 μm. (**b**) Quantification of the CD206⁺ M2-like/CD86⁺ M1-like macrophage ratio (n = 5; mean ± SEM). (**c**) Representative flow cytometry plots showing CD14, CD86, and CD206 staining under mono-culture and co-culture conditions. (**d**) Representative histograms of CD86 (Pacific Blue) and CD206 (APC) expression, gating M1-like and M2-like macrophages, respectively. (**e–g**) Quantification under mono-culture and co-culture conditions (n = 3; mean ± SEM). (**e**) percentage of CD14⁺CD86⁺ M1-like macrophages, (**f**) percentage of CD14⁺CD206⁺ M2-like macrophages, and (**g**) ratio of CD14⁺CD206⁺ to CD14⁺CD86⁺ macrophages.

**Fig. S5.** **Cellular compositional changes in carious dental pulp.** (**a**) Dot plots depicting expression of cell type marker genes. (**b**) UMAP visualization of *THY1* expression in dental pulp cell clusters. (**c**) Stacked bar graph showing the relative proportion (%) of each cell type across healthy, moderate caries, and severe caries groups. (**d**) Bar graph of total number of interactions and signaling strength.

**Fig. S6.** **Cell–cell communication analysis in the dental pulp.** (**a**) Heatmap showing the four centrality indicators of the CXCL signaling pathway. (**b**) The inferred VEGF signaling networks. Circle sizes are proportional to the number of cells in each cell group and edge width represents the communication probability. (**c**) Heatmap showing the four centrality indicators of the VEGF signaling pathway. (**d**) The inferred FN1 signaling networks. Circle sizes are proportional to the number of cells in each cell group and edge width represents the communication probability. (**e**) Heatmap showing the four centrality indicators of the FN1 signaling pathway.

**Fig. S7. Functional inhibition of fibronectin–mediated pro-angiogenic signaling reduces angiogenic activity and cell survival.** (**a**) Quantitative analysis of angiogenic parameters, including the numbers of junctions, segments, and meshes, using the Angiogenesis Analyzer in HUVECs cultured with conditioned medium derived from co-cultures with or without RGD peptide pretreatment of macrophages (MPs; Co + RGD) (n = 4–6; mean ± SEM). (**b**) Quantification of cleaved-caspase-3 signal intensity in conditioned medium from co-culture or Co + RGD conditions (n = 7; mean ± SEM). (**c**) Quantitative analysis of angiogenic parameters in conditioned medium from co-culture, SB-treated co-culture (Co + SB), or AAL-treated co-culture (Co + AAL) (n = 4; mean ± SEM). (**d**) Quantification of cleaved-caspase-3 signal intensity in conditioned medium from co-culture, Co + SB, or Co + AAL conditions (n = 4; mean ± SEM). *Nb. Junctions = number of junctions; Nb. Segments = number of segments; Nb. Meshes = number of meshes. SB (SB225002); CXCR2 inhibitor, AAL (AAL-993); VEGFR inhibitor. Statistical analysis was performed using Student’s t-test.

**Fig. S8. Histological comparison of pulp-dentin-like tissue formation and apical closure after revascularization.** (**a–d**) Fibronectin-treated root. (**a**) μCT image showing the analyzed root region, including the apical closure area. (**b**) Low-magnification H&E image showing organized pulp-dentin-like tissue formation, abundant blood vessels within the pulp-like tissue, and apical hard tissue closure. The small and large boxed regions correspond to the higher-magnification images shown in (c) and (d), respectively. (**c**) Higher-magnification H&E image of the dentin-pulp interface showing newly formed dentin-like tissue and an organized odontoblast-like cell layer adjacent to the original dentin. (**d**) Higher-magnification H&E image of the apical region showing hard tissue closure with multiple channel-like structures, resembling the apical architecture of mature canine teeth. (**e–h**) Control root. (**e**)  μCT image showing incomplete apical closure with thin root dentin walls. (**f**) Low-magnification H&E image showing loosely organized amorphous fibrous tissue within the canal space, with fewer blood vessels and no evident apical hard tissue closure. The small and large boxed regions correspond to the higher-magnification images shown in (g) and (h), respectively. (**g**) Higher-magnification H&E image showing the absence of a well-organized odontoblast-like cell layer and limited dentin-like hard tissue deposition adjacent to the original dentin. (**h**) Higher-magnification H&E image of the apical region showing fibrous connective tissue without evident apical hard tissue closure. Scale bars: as indicated.

**Fig. S9. Immunofluorescence analysis of macrophage populations in the odontoblastic cell layer and stromal region.** (**a**) Representative immunofluorescence images showing CD14 (green), CD163 (magenta), and nuclei (Hoechst; blue) in the odontoblastic cell layer of beagle teeth. Scale bars: 50 μm. (**b**) Representative immunofluorescence images showing CD14 (green), CD163 (magenta), and nuclei (Hoechst; blue) in the stromal region of beagle teeth. Scale bars: 50 μm. (**c**) Percentage of CD14⁺CD163⁺ cells (n = 4; mean ± SEM).

**Fig. S10. Fibronectin-mediated macrophage-DPSC crosstalk driving angiogenic niche formation in the regenerating pulp-dentin complex.** Fibronectin-mediated crosstalk between macrophages and DPSCs enhances the expression of pro-angiogenic factors, such as angiogenic chemokines and VEGF. This interaction promotes endothelial cell differentiation and angiogenic niche formation, which supports neovascularization and contributes to the regeneration of the damaged pulp-dentin complex. Created with BioRender.com.

**Supplemental Tables**

**Table S1. The list of primers used in this study.**

| Target Gene | Species | Primer sequence (5’ - 3') | |
| --- | --- | --- | --- |
| *GAPDH* | human | Forward | TCC TGC ACC ACC AAC TGC TT |
|  |  | Reverse | TGG CAG TGA TGG CAT GGA C |
| *CXCL2* | human | Forward | GGC AGA AAG CTT GTC TCA ACC C |
|  |  | Reverse | CTC CTT CAG GAA CAG CCA CCA A |
| *CXCL3* | human | Forward | TTC ACC TCA AGA ACA TCC AAA GTG |
|  |  | Reverse | TTC TTC CCA TTC TTG AGT GTG GC |
| *CXCL5* | human | Forward | CAG ACC ACG CAA GGA GTT CAT C |
|  |  | Reverse | TTC CTT CCC GTT CTT CAG GGA G |
| *CXCL6* | human | Forward | GGG AAG CAA GTT TGT CTG GAC C |
|  |  | Reverse | AAA CTG CTC CGC TGA AGA CTG G |
| *CXCL8* | human | Forward | GAG AGT GAT TGA GAG TGG ACC AC |
|  |  | Reverse | CAC AAC CCT CTG CAC CCA GTT T |
| *VEGFA* | human | Forward | TTG CCT TGC TGC TCT ACC TCC A |
|  |  | Reverse | GAT GGC AGT AGC TGC GCT GAT A |
| *ITGα1* | human | Forward | GGT TCC TAC TTT GGC AGT ATT |
|  |  | Reverse | AAC CTT GTC TGA TTG AGA GCA |
| *ITGα2* | human | Forward | GGA ACG GGA CTT TCG CAT |
|  |  | Reverse | GGT ACT TCG GCT TTC TCA TCA |
| *ITGα5* | human | Forward | TGC AGT GTG AGG CTG TGT ACA |
|  |  | Reverse | GTG GCC ACC TGA CGC TCT |
| *ITGα11* | human | Forward | GGA GGA AGA CTT GCG TCG |
|  |  | Reverse | CAC AGG TTC CCC AGT AGA TG |
| *ITGα3* | human | Forward | AAG GGA CCT TCA GGT GCA |
|  |  | Reverse | TGT AGC CGG TGA TTT ACC AT |
| *ITGαV* | human | Forward | AAT CTT CCA ATT GAG GAT ATC AC |
|  |  | Reverse | AAA ACA GCC AGT AGC AAC AAT |
| *ITGβ1* | human | Forward | GAA GGG TTG CCC TCC AGA |
|  |  | Reverse | GCT TGA GCT TCT CTG CTG TT |
| *ITGβ3* | human | Forward | CCG TGA CGA GAT TGA GTC A |
|  |  | Reverse | AGG ATG GAC TTT CCA CTA GAA |
| *ITGβ5* | human | Forward | GGA GCC AGA GTG TGG AAA CA |
|  |  | Reverse | GAA ACT TTG CAA ACT CCC TC |
| *ITGβ8* | human | Forward | AAT TTG GTA GTG GAA GCC TAT C |
|  |  | Reverse | GTC ACG TTT CTG CAT CCT TC |
| *FN1* | human | Forward | ACC AAC CTA CGG ATG ACT CG |
|  |  | Reverse | GCT CAT CAT CTG GCC ATT TT |

**Table S2. Antibodies for protein analysis.**

| Antigen | Source | Dilution | Commercial Source | Cat. No. |
| --- | --- | --- | --- | --- |
| Primary Antibody | | | | |
| CXCL6 | Rabbit | 1:1000 | Cell Signaling Technology | 73201 |
| CXCL8 | Rabbit | 1:1000 | Cell Signaling Technology | 94407 |
| VEGF-A | Rabbit | 1:1000 | Cell Signaling Technology | 50661 |
| Thy1/CD90 | Rabbit | 1:200 | Cell Signaling Technology | 13801 |
| CD31 (PECAM-1) | Mouse | 1:200 | Cell Signaling Technology | 3528 |
| Cleaved Caspase 3 | Rabbit | 1:400 | Cell Signaling Technology | 9661 |
| β-actin | Mouse | 1:3000 | Santa Cruz Biotechnology | sc-47778 |
| NF-κB (p65) | Rabbit | 1:200 | Santa Cruz Biotechnology | sc-372 |
| Integrin α5 | Rabbit | 1:200 | Novus | 4705 |
| Thy-1 | Sheep | 1:200 | Novus | AF2067 |
| Fibronectin | Mouse | 1:500 | BD Bioscience | 610077 |
| CD14, FITC | Human | 1:50 | Biolegend | 325603 |
| CD68 | Rabbit | 1:200 | Dako | M0814 |
| MSR1 | Rabbit | 1:200 | Origene | TA336699 |
| FAK | Rabbit | 1:1000 | Santa Cruz Biotechnology | sc-557 |
| Phospho-FAK (Tyr397) | Rabbit | 1:1000 | Invitrogen | 700255 |
| CD86, Pacific Blue | Human | 1:100 | Biolegend | 305418 |
| CD86, APC | Human | 1:100 | Biolegend | 305412 |
| CD206, FITC | Human | 1:100 | Biolegend | 321104 |
| CD206, APC | Human | 1:100 | Biolegend | 321110 |
| CD45, eFluor™ 450 | Dog | 1:100 | Invitrogen | 48-5450-42 |
| CD14, FITC | Dog | 1:100 | Invitrogen | MA1-82074 |
| CD163 | Dog | 1:100 | Invitrogen | BS-2527R |
| Immunoperoxidase | | | | |
| IgG (H&L) anti-rabbit | Mouse | 1:5000 | Santa Cruz Biotechnology | sc-2357 |
| IgG (H&L) anti-mouse | Goat | 1:5000 | KOMA Biotech | K0211589 |
| Immunofluorescence | | | | |
| anti-rabbit (Alexa 488) | Goat | 1:500 | Invitrogen | A-11008 |
| anti-mouse (Alexa 488) | Goat | 1:500 | Invitrogen | A-11001 |
| anti-rabbit (Alexa 594) | Goat | 1:500 | Invitrogen | A-11012 |
| anti-rabbit (Alexa 594) | Donkey | 1:500 | Invitrogen | A-21203 |
| anti-sheep (DyLight 650) | Rabbit | 1:50 | Invitrogen | SA5-10057 |

**Materials and Methods**

**Cell culture**

Human macrophages (MPs) were generated from U937 monocytes (Korean Cell Line Bank, KCLB 21593.1) by treatment with 100 ng/mL phorbol 12-myristate 13-acetate (PMA; Sigma, P8139) for 2 days. U937 cells were maintained in RPMI 1640 medium (Welgene, LM011-07) supplemented with 10% fetal bovine serum (FBS; Welgene, S001-07). Dental pulp stromal cells (DPSCs; Lonza, PT-5025) were cultured in Dulbecco’s Modified Eagle’s Medium (DMEM; Welgene, LM001-05) containing 10% FBS and 1% penicillin–streptomycin (Gibco, 15140-122). For co-culture experiments, DPSCs were seeded in 96-well or 6-well plates one day prior to co-culture, after which PMA-differentiated MPs were added and co-cultured for 1–3 days. Human umbilical vein endothelial cells (HUVECs; PromoCell, C-12206) were maintained in Endothelial Cell Growth Medium 2 (PromoCell, C-22011) supplemented with the corresponding pack, excluding fibroblast growth factor (FGF) and vascular endothelial growth factor (VEGF). Murine RAW264.7 macrophages (ATCC, TIB-71) were used for gene manipulation studies and cultured in RPMI 1640 medium with 10% FBS under standard conditions.

**Reagents and plasmids**

The chemical reagents and plasmids used in this study were as follows: 100 ng/mL phorbol 12-myristate 13-acetate (PMA; Sigma, P8139), Texas Red-X Phalloidin (Thermo Fisher Scientific, T7471), fibronectin (2–100 µg/mL; Sigma, F1141), type I bovine collagen (3 µg/mL; PureCol®, Cellink, 5005), collagenase type I (25 µg/mL; Worthington Biochemical, nc9482366), and RGD peptide (10–100 µg/mL; Tocris). AAL-993 (VEGFR inhibitor; 100 nM; MCE; HY-19986) and SB225002 (CXCR2 inhibitor; 30 nM; Sigma; SML0716) were used. Macrophage depletion was performed using clodronate-encapsulated liposomes (Encapsula Nanosciences, CLD-8901). The pSIRV-NF-κB-eGFP plasmid (Addgene, #118093) was purchased from Addgene. The antibodies used in this study are listed in Table S2.

**CRISPR/Cas9-mediated gene editing**

To generate *ITGA5* knockout cells, a guide RNA with the sequence 5'-GGGCTTCAACCTAGACGCGGAGG-3' was designed (Bioneer). The murine macrophage cell line RAW264.7 was transfected with CRISPR plasmids (sgRNA and Cas9 expression vectors) using AccuFect™ transfection reagent (Bioneer) according to the manufacturer's protocol. *ITGA5* knockout efficiency was validated by quantitative PCR (qPCR) analysis.

**Live-cell imaging**

Live-cell imaging was performed using Cytation5 (Agilent Technologies, Inc.) at 15-minute intervals under laser autofocus conditions. Phase-contrast imaging was used to monitor morphological changes, while fluorescence imaging tracked fluorescence intensity changes in real time. Fluorescence intensity was quantified by tracking individual cells in each image using either the Time-lapse Analyzer or the ROI Measurement function in the Fiji software plugin.

**Fibronectin coating assay**

To coat the culture plates, fibronectin (Sigma, F1141) was diluted in sterile water to a final concentration of 2–100 µg/mL. The fibronectin solution was applied to the plates and incubated at 37 °C in 5% CO₂ for 1 h. Excess solution was removed, and the plates were then air-dried for an additional hour at room temperature before macrophages and medium were added.

**RNA isolation and quantitative real-time polymerase chain reaction (qRT-PCR)**

Total RNA was isolated from cells using TRIzol reagent (Invitrogen, 15596026) following the manufacturer’s protocol. cDNA synthesis was performed with TOPscript RT DryMIX (Enzynomics, RT200) using human gene-specific primers (primer sequences listed in Table S1). Quantitative PCR (qPCR) was carried out using SYBR Green qPCR Master Mix (Applied Biosystems, 4309155). Relative gene expression was analyzed using the ΔΔCt method, with GAPDH serving as the internal reference gene for normalization.

**Immunoblot analysis**

The cells were lysed in RIPA lysis buffer (GenDEPOT, R4200-010) containing protease inhibitor cocktail (GenDEPOT, P3100) and phosphatase inhibitor cocktail solution (GenDEPOT, P3200) on ice. Cell lysates were boiled in SDS sample buffer at 98 °C for 5 min, separated on a 10% sodium dodecyl sulfate-polyacrylamide gel, and transferred to a nitrocellulose membrane using an iBlot 2 Dry Blotting System (Invitrogen, IB23001). The membranes were blocked with 5% skim milk (BD Difco, 232100) in Tris-buffered saline with 0.1% Tween-20 (TBS-T; CURE BIO, T0209) for 1 h, and incubated with specific primary antibodies at 4 °C overnight. After washing with TBS-T, the membranes were incubated with horseradish peroxidase-conjugated (HRP) secondary antibodies at 37 °C for 1 h. Signal detection was performed using enhanced chemiluminescence reagent (Thermo Fisher Scientific, 34579) and visualized using a Fusion Fx6 imager (Witec AG).

**Supernatant protein precipitation**

The culture supernatants (1 mL each) were subjected to protein precipitation using 10% trichloroacetic acid (TCA), prepared by mixing 1 volume of 100% TCA stock solution with 9 volumes of the sample. The 100% (w/v) TCA stock was prepared by dissolving 500 g of TCA (Sigma, T6399) in 500 mL of distilled water and stored at room temperature. For precipitation, 100 μL of the TCA stock was added to 1 mL of culture supernatant in 1.5 mL microcentrifuge tubes. Samples were incubated on ice at 4 °C for 10 min, followed by centrifugation at 14,000 rpm for 5 min at 4 °C. The resulting protein pellets were washed twice with 200 μL of ice-cold acetone, each followed by centrifugation at 14,000 rpm for 5 min. Pellets were dried by placing the tubes in a 95 °C heat block for 5–10 min to evaporate residual acetone. Dried pellets were rehydrated using 20–100 μL of 0.2 N NaOH for 2–5 min. The pellets were then washed with ice-cold DPBS and centrifuged at 14,000 rpm for 5 min at 4 °C. The final pellets were lysed in RIPA lysis buffer, and the lysates were clarified by centrifugation at 14,000 rpm for 5 min at 4 °C. TCA-precipitated supernatants and clarified cell lysates were mixed with SDS-PAGE sample buffer and boiled at 95 °C for 5–10 min for protein denaturation prior to electrophoresis.

**Flow cytometry assay**

To purify heterogeneous cell populations within each group, fluorescence-activated cell sorting (FACS) was employed. Mono- and co-cultured cells were detached using trypsin-EDTA and processed separately for analysis. Cells were incubated with 5% FBS for 30 min at room temperature to block nonspecific binding. After washing with phosphate-buffered saline (PBS), cells were labeled with primary antibodies against CD14 (macrophage marker) and Thy-1 (DPSC marker). Next, cells were stained with FITC- or APC-conjugated secondary antibodies (1:50 dilution) in the dark on ice for 30 min, followed by two additional PBS washes. Stained cells were sorted using a FACS Aria III cell sorter (BD Biosciences). Four distinct populations were isolated for downstream gene expression analysis: Thy-1⁺ cells from mono-culture, Thy-1⁺ cells from co-culture, CD14⁺ cells from mono-culture, and CD14⁺ cells from co-culture (Fig. S2a). To assess macrophage polarization under mono- and co-culture conditions, we characterized established markers of M1-like and M2-like macrophages (Fig. S4). Following Fc receptor blocking, cells were stained with 7-AAD viability dye (Biolegend, 420403), FITC-conjugated anti-human CD14, Pacific Blue–conjugated anti-human CD86, and APC-conjugated anti-human CD206. All stained cells were analyzed on a BD FACSVerse™ flow cytometer (BD Biosciences), and the data were processed with FlowJo software (TreeStar).

**Bulk RNA-sequencing**

To evaluate total RNA integrity, samples were analyzed using the RNA ScreenTape system (Agilent Technologies, 5067-5576). Only RNA samples with a RIN value greater than 7.0 were selected for library preparation. For each sample, 1 µg of total RNA was used to generate a library using the Illumina TruSeq Stranded mRNA Sample Preparation Kit (Illumina, RS-122-2101). Polyadenylated mRNA was isolated using poly-T oligo-attached magnetic beads and subsequently fragmented by exposure to divalent cations under elevated temperature. First-strand cDNA synthesis was performed using SuperScript Ⅱ Reverse Transcriptase (Invitrogen, 18064014) and random hexamer primers, followed by second-strand synthesis using DNA polymerase I, RNase H, and dUTP incorporation. The resulting double-stranded cDNA underwent end repair, addition of a single ‘A’ nucleotide, and ligation of sequencing adapters. The libraries were then purified and amplified by PCR to generate the final cDNA libraries. Library quantification was conducted using the KAPA Library Quantification Kit (Kapa Biosystems, KK4854) according to the manufacturer's qRT-PCR protocol, and quality was assessed using the D1000 ScreenTape system (Agilent Technologies, 5067-5582). Indexed libraries were sequenced on the Illumina NovaSeq platform (2 × 100 bp, paired-end) by Macrogen Inc.

**Single-cell RNA sequencing data analysis**

Single-cell RNA sequencing data were processed and analyzed using Seurat (v4.3.0). Gene expression data were normalized and scaled using the SCTransform method. Dimensionality reduction was initially performed using Principal Component Analysis (PCA), followed by batch effect correction across samples from different conditions (healthy teeth, mild caries, and severe caries) using the Harmony package (v1.0) within the Seurat framework. After data integration, Uniform Manifold Approximation and Projection (UMAP) was applied for further dimensionality reduction. Cluster identification was conducted using Seurat’s graph-based clustering approach. Cell type annotation was manually performed based on the expression profiles of known cell type-specific marker genes, in conjunction with relevant literature. Visualization of marker gene expression was carried out using ggplot2 (v3.4.4).

**Cell-cell communication analysis**

Intercellular communication networks were analyzed using the CellChat package (v1.6.1) following the standard pipeline. Normalized expression data from Seurat were imported into CellChat and subjected to standard preprocessing. Ligand–receptor interactions were inferred by calculating communication probabilities and performing permutation-based significance tests. Significant interactions were aggregated to construct communication networks based on interaction strength and connectivity degree. To assess changes in signaling dynamics, information flow of signaling pathways was compared among healthy, mild caries, and severe caries conditions. Euclidean distances between shared signaling pathways were computed to quantify pathway-level divergence across experimental groups.

**Confocal and super-resolution imaging of cells**

Cells were seeded at 70% confluence in 96-well plates (Ibidi GmbH, 89626). DPSCs were fixed with 4% paraformaldehyde (BioPrince, BPP-9004) at 37 °C for 20 min and washed three times with PBS containing 0.1% (v/v) Tween-20 (Sigma-Aldrich, P9416). After fixation, the cells were permeabilized with 0.1% Triton X-100 in PBS for 30 min and washed three times with PBST. Samples were blocked with 10% FBS for 1 h at room temperature. Following three additional washes, the primary antibody was applied and incubated at 4 °C for 24 h in a humidified chamber. The samples were then incubated with secondary antibody (diluted in PBS) at 37 °C for 1 h in the dark, followed by nuclear counterstaining with DAPI (Sigma-Aldrich, D9542). After the final three washes with PBST, fluorescence imaging was performed using an LSM 800 confocal laser scanning microscope (Carl Zeiss, Jena). Three-dimensional image reconstruction of Airyscan confocal and lattice SIM images was carried out using Imaris 9.8 software (Bitplane). For super-resolution live-cell imaging, lattice SIM images were acquired every 4 seconds using a Zeiss Elyra 7 system equipped with Lattice SIM and an alpha Plan-Apochromat 63×/1.46 NA oil immersion objective.

**Scanning electron microscopy (SEM) sample preparation and imaging**

Cells were fixed with 2.5% glutaraldehyde for 2 hours at 4 °C. After washing with PBS, the samples were post-fixed with 1% osmium tetroxide on ice for 1 h. The samples were then dehydrated through a graded ethanol series and chemically dried using hexamethyldisilazane (HMDS). Following drying, the samples were coated with platinum using an ion sputter coater (Quorum Technologies, Q150TS) at 30 mA for 160 seconds. SEM imaging was performed using an Apreo S scanning electron microscope (Thermo Scientific) at an accelerating voltage of 10 kV, at Seoul National University School of Dentistry and Dental Research Institute. SEM specimen preparation was performed in accordance with the NICEM protocol.

**Animal Housing, Husbandry, and Perioperative Care**

All animal procedures were performed in accordance with the approved Institutional Animal Care and Use Committee (IACUC) protocol of Yonsei University Health System (Approval No. P-2021-0130) and Cronex (Approval No. 202603004). Animals were acclimatized for at least 7 days before the experimental procedures and were housed in the institutional large-animal facility under veterinary supervision. Dogs were housed individually, with visual and limited social contact through cage windows, and were provided standard food and water according to institutional husbandry procedures. Environmental conditions, cage sanitation, feeding, and routine health checks were managed by trained animal-care staff.

All dental procedures were performed under general anesthesia. Anesthesia was induced with alfaxalone and maintained with isoflurane after endotracheal intubation. Perioperative analgesia and antibiotic prophylaxis were provided according to the approved veterinary protocol, including meloxicam for pain control and cefazolin for antibiotic coverage. Animals were monitored during the perioperative and postoperative periods for general condition, food intake, wound healing, signs of pain or distress, and procedure-related complications. Humane endpoints were predefined in the approved protocol and included severe or persistent pain, infection, marked reduction in food intake, abnormal behavior, or any condition requiring veterinary intervention.

At the planned experimental endpoint, animals were euthanized under deep general anesthesia according to the approved protocol. Deep anesthesia was confirmed before intravenous potassium chloride administration. No unexpected adverse events were observed during the experimental period.

**μCT analysis**

Root specimens were scanned using a μCT system (Skyscan 1173; Bruker, Kontich, Belgium). Radiographic projections were acquired at 130 kV and 60 µA with a 500 ms exposure time, a 1.0 mm-thick aluminum filter, and a 5 μm pixel size. The acquired images were reconstructed using NRecon software version 1.7.4.6 (Bruker, Kontich, Belgium) and analyzed using CtAn software version 1.19.4.0 (Bruker, Kontich, Belgium).

**Histological preparation**

Root specimens were fixed in 10% neutral buffered formalin and subsequently decalcified for 2 weeks in 10% ethylenediaminetetraacetic acid (EDTA, pH 7.4) at 42°C. The decalcified specimens were dehydrated sequentially through graded ethanol solutions (70%, 95%, and 100%) followed by xylene treatment. Samples were then embedded in paraffin and sectioned at 3 μm thickness using a microtome (RM2255; Leica, Wetzlar, Germany). The sectioned slides were deparaffinized in xylene and rehydrated through a graded series of ethanol solutions (100%, 95%, and 70%) followed by distilled water. Hematoxylin and eosin (H&E) staining was performed for morphological evaluation. Histological images were captured using a light microscope (BX51; Olympus, Tokyo, Japan).

**Immunofluorescence staining of formalin-fixed paraffin-embedded tissue**

Human and beagle teeth were decalcified in 10% ethylenediaminetetraacetic acid (EDTA; pH 7.4) for 1 month with daily solution changes. Decalcified tissues were washed, dehydrated, and embedded in paraffin. Paraffin blocks were sectioned at 4 μm thickness and mounted on silane-coated glass microscope slides (Muto Pure Chemicals Co., Ltd., 5116-20F). For immunofluorescence staining, paraffin-embedded sections were deparaffinized and rehydrated through a graded ethanol series. Heat-mediated antigen retrieval was performed using Tris/EDTA buffer (pH 9.0) according to standard protocols. Sections were blocked with 5% bovine serum albumin (Sigma-Aldrich) for 1 h at room temperature to reduce nonspecific binding. Primary antibodies were applied, and sections were incubated overnight at 4 °C in a humidified chamber. After three washes with PBS, fluorescently conjugated secondary antibodies and Hoechst (nuclei) diluted in PBS were applied and incubated for 1 h at room temperature in the dark. Following three additional washes with PBS containing 0.1% Tween-20 (PBST), the sections were mounted using an appropriate mounting medium and visualized with an LSM 800 confocal laser scanning microscope (Carl Zeiss, Jena).

**Hematoxylin and eosin staining**

Following decalcification, specimens were embedded in paraffin and longitudinally sectioned at 3 μm thickness using a microtome. Sections were mounted on slides and heated at 55 °C for deparaffinization. Samples were deparaffinized through four sequential 5-min changes in xylene, followed by rehydration in decreasing concentrations of ethanol (100%, 95%, 80%, and 70%) for 2 min each. Slides were washed in running distilled water for 1 min and stained with Harris hematoxylin-I for 5 min, followed by a 1-min wash under running tap water. Sections were briefly differentiated in ammonia water for 30 s and washed with tap water for 3 min. Subsequently, sections were counterstained with Eosin Y for 2 min 30 s, followed by dehydration through graded ethanol (95%–100%). Slides were cleared in xylene and mounted with appropriate mounting medium.

**RNA interference**

Small interfering RNA (siRNA) transfection was performed using Dharmacon ON-TARGETplus siRNA SMART pools (Dharmacon, Lafayette, USA). The following siRNAs were used: human *FN1* (L-009853-00-0010, Dharmacon), ON-TARGET plus non-targeting control siRNAs (D-001810-01-20, Dharmacon). Cells were plated in 12-well plates and transfected at a final concentration of 25 nM using Lipofectamine 2000 (11668-019, Invitrogen). Lipofectamine 2000 and siRNA were diluted separately in Opti-MEM® (TTFS-31985070, Gibco BRL) at 37 °C for 5 min. The mixture was then incubated at RT for 25 min. To each well, this transfection medium was added, followed by incubation at 37 °C with 5% CO_2_ for 3 h. After removing the transfection medium, the cells were incubated with fresh medium for 48 h. Knockdown efficiency was verified by qRT-PCR.
